# A rapid review of interventions to improve medicine self‐management for older people living at home

**DOI:** 10.1111/hex.13729

**Published:** 2023-03-14

**Authors:** Giorgia Previdoli, V‐Lin Cheong, David Alldred, Justine Tomlinson, Savi Tyndale‐Biscoe, Jonathan Silcock, Daniel Okeowo, Beth Fylan

**Affiliations:** ^1^ Yorkshire Quality and Safety Group Bradford Teaching Hospitals NHS Foundation Trust Bradford United Kingdom; ^2^ Medicines Management & Pharmacy Services Leeds Teaching Hospitals NHS Trust Leeds United Kingdom; ^3^ Faculty of Medicine and Health, School of Healthcare University of Leeds Leeds United Kingdom; ^4^ Faculty of Life Sciences, School of Pharmacy and Medical Sciences University of Bradford Bradford United Kingdom; ^5^ Bradford Teaching Hospital Foundation Trust Bradford United Kingdom

**Keywords:** medication management, medication safety, older people, patient safety, rapid review, Resilient Health Care

## Abstract

**Background:**

As people age, they are more likely to develop multiple long‐term conditions that require complicated medicine regimens. Safely self‐managing multiple medicines at home is challenging and how older people can be better supported to do so has not been fully explored.

**Aim:**

This study aimed to identify interventions to improve medicine self‐management for older people living at home and the aspects of medicine self‐management that they address.

**Design:**

A rapid review was undertaken of publications up to April 2022. Eight databases were searched. Inclusion criteria were as follows: interventions aimed at people 65 years of age or older and their informal carers, living at home. Interventions needed to include at least one component of medicine self‐management. Study protocols, conference papers, literature reviews and articles not in the English language were not included. The results from the review were reported through narrative synthesis, underpinned by the Resilient Healthcare theory.

**Results:**

Database searches returned 14,353 results. One hundred and sixty‐seven articles were individually appraised (full‐text screening) and 33 were included in the review. The majority of interventions identified were educational. In most cases, they aimed to improve older people's adherence and increase their knowledge of medicines. Only very few interventions addressed potential issues with medicine supply. Only a minority of interventions specifically targeted older people with either polypharmacy, multimorbidities or frailty.

**Conclusion:**

To date, the emphasis in supporting older people to manage their medicines has been on the ability to adhere to medicine regimens. Most interventions identify and target deficiencies within the patient, rather than preparing patients for problems inherent in the medicine management system. Medicine self‐management requires a much wider range of skills than taking medicines as prescribed. Interventions supporting older people to anticipate and respond to problems with their medicines may reduce the risk of harm associated with polypharmacy and may contribute to increased resilience in the system.

**Patient or Public Contribution:**

A patient with lived experience of medicine self‐management in older age contributed towards shaping the research question as well as the inclusion and exclusion criteria for this review. She is also the coauthor of this article. A patient advisory group oversaw the study.

## 1 INTRODUCTION

The global population is ageing rapidly. The Population Division of the United Nations estimated that the percentage of people aged 65 years of age or older worldwide has grown from 6% in 1990 to 9% in 2019, and is projected to increase further to 16% by 2050.^1^ In the United Kingdom, by 2039, nearly one in every four (23.9%) people will be aged 65 years and older.^2^ As people age, the prevalence of multimorbidity, the coexistence of two or more chronic conditions, also increases,^3^, ^4^ which impacts both quality of life^5^ and the cost of healthcare.^6^

With multimorbidity comes polypharmacy, most commonly defined as the concurrent use of 5 or more medicines.^7^ A large European cohort study found that 32% of citizens aged 65 years or older were taking five or more medicines.^8^ A cohort study in Sweden found that 44% of those aged 65+ took at least 5 medicines and 12% took 10 medicines or more^9^, and a recent US cross‐sectional study of patients 65 years of age and older found that 37% were prescribed five or more medicines.^10^ In the United Kingdom, a Scottish study found that 35% of those aged 85 years and older take more than 10 medicines,^11^ while the most recent data on medicines prescribed within the National Healthcare Service (NHS) in England indicated that, by the age of 80, a third of the population takes eight or more medicines.^12^

Polypharmacy has been linked to a range of negative outcomes in older people, including drug‐related problems, adverse drug events, impact on physical and cognitive function, hospitalization and mortality.^13^ Managing medicines poses multiple challenges to older patients and those who support them. Older patients, for example, have been found to face difficulties in filling prescriptions, especially after an unplanned hospitalization, in reading and understanding medicines’ instructions, in opening containers and retrieving medications, in swallowing pills, in taking the right medicines at the right time and in detecting and reporting adverse reactions.^14^

Moreover, older patients prescribed many medicines are likely to have to manage a complex medicine regimen, often with no or limited support. According to George and colleagues,^15^ regimens are complex when medicines come in different forms (e.g., pills, injections, drops), have different dosing frequencies and come with additional instructions that guide administration. Evidence from a Swedish cohort study has shown that, in patients aged 60 years and older, medication regimen complexity is associated with increased mortality.^16^

Among the older population, patients living with frailty have been found to be particularly vulnerable to adverse events associated with complex medicine regimens.^17^ Described as an ageing‐related process in which multiple body systems gradually lose their in‐built reserves and become increasingly vulnerable to relatively minor stressor events,^18^ frailty is estimated to affect around 10% of people over 65 years.^19^

Thus, there is a clear need to improve older patients’ abilities to self‐manage their medicines as well as their abilities to detect and respond to problems, to prevent deterioration.

Bailey and colleagues define medicine self‐management as ‘the extent to which a patient takes medication as prescribed, including not only the correct dose, frequency and spacing, but also its continued, safe use over time’.^20^ This requires a range of knowledge, skills and behaviours and patients must follow six steps to safely and effectively use their medication in primary care: (1) filling (getting hold of the medicines prescribed at the right time), (2) understanding, (3) organizing, (4) taking, (5) monitoring and (6) sustaining. More recently, Howell and colleagues^21^ focused on the functional skills required by patients to manage medicine preparation and administration. In their realist synthesis, Maidment and colleagues^22^ looked at how older people, family carers and healthcare professionals (HCPs) engage in medicine management at five different stages: (1) identifying problems, (2) getting diagnosis/medications, (3) starting/stopping medications, (4) continuing to take medications and (5) reviewing medications. Finally, Cheraghi Sohi, Schafheutle and their colleagues^23^, ^24^ looked at medicine self‐management as a workload carried out by patients, often with support from their family and their informal network,^25^ in four main areas: medication articulation work, surveillance work, emotional work and informational work.

While managing many medicines poses multiple challenges, older patients and patients with long‐term chronic conditions are able to overcome difficulties and prevent harm caused by medicines.^26^ Adopting a Safety II (Resilient Healthcare)^27^ approach, Fylan and colleagues^28^ looked at how cardiology patients managed their medicines after discharge from hospital. They found that some patients contributed to medicine management system resilience through monitoring and responding to supply problems, anticipating discrepancies and notifying HCPs of errors in documentation. Some anticipated problems with their own adherence and put mitigating strategies in place. Tomlinson and colleagues found that some patients and their support networks were proactive, for example, in seeking additional information on new medicines introduced or facilitating communications between hospital and pharmacy to mitigate disruptions in their supply.^26^

Whilst such observational studies have identified some people's abilities to implement safety strategies, there is a lack of evidence concerning the range of interventions that support safe medicine self‐management activities, such as adherence to the regimen, knowledge of medicines and condition, supply management and monitoring effects.^29^ Whilst there is a wealth of evidence about how healthcare staff can reduce the risk of adverse drug events, for example, through deprescribing, medication review and staff training,^30^, ^31^ there is less focus on developing interventions to improve the role that patients themselves can play in the safety of medicines. Interventions that build on enhancing the way some older people safely self‐manage their medicines at home may have a positive impact on their health outcomes. This rapid review, therefore, aims to explore the range and nature of interventions that support medicine self‐management for older people. We wish to understand the self‐management components that they target and the abilities they aim to support patients to develop, so that gaps in patient support can be identified and addressed.

## 2 METHODS

A rapid review^32^ was undertaken to inform intervention development.^33^ A rapid process was chosen as a pragmatic approach to generate evidence to inform empirical research and codesign activities in a timely way. Only published papers in English were included and no searches were conducted for grey literature or citations.^32^ Measures were adopted to mitigate the risk of excluding relevant papers and to maintain consistency in applying the inclusion criteria among the team. The measures included testing and refining the screening checklist using a limited number of papers and multiple researchers screening a sample of excluded papers at each stage.^32^, ^33^, ^34^

### 2.1 Search strategy and selection criteria

Searches were run on 11 April 2022. The following databases were searched: CINAHL, MEDLINE, EMBASE, PUBMED, PSYCHINFO, COCHRANE and Social Care Online. The search terms were identified by a multidisciplinary team including experts in patient safety, social researchers, pharmacists and a patient and public representative. A health subject librarian helped to translate each of five concepts into MeSH terms, key words and combination of key words and phrases. The five search concepts were combined in search using the Boolean operator AND (see Supporting Information: Appendix 1 MeSH terms and Key words). Grey literature was not searched due to time constraints and the objectives of the review. The review addressed the following research question: ‘What interventions have been developed to support community‐dwelling older people to self‐manage their medicines?’.

The Medical Subject Headings (MeSH terms) and key words selected addressed five different concepts: (1) ‘older age’, (2) ‘living in the community’, (3) living with or without support from ‘informal carers’, (4) ‘self‐management’ and (5) ‘medicines’. Two examples of the strings used to search articles on databases are reported in the see Supporting Information: Appendix 1 (MeSH terms and Key words).

To be included in the review, studies needed to focus on medicine self‐management and specifically target an older population (65 years old or older). At full‐text screening, papers were retained if age of 65 years and older was one of the inclusion criteria or if the results reported a prevalence of participants aged 65 years or older (or informal carers of patients in the same age group).

Articles were excluded if they were an editorial, a research protocol, a thesis, a conference paper or a literature review. Only articles written in English were considered. Articles were excluded if they focused on improving medicine management at care transitions because there is already a rich literature around interventions to support older people at hospital discharge or moving between different care settings.^35^ Our focus in conducting this review was on interventions addressing the less frequently explored topic of self‐management of medicines and its components (e.g., adherence, knowledge, supply management and monitoring effects)^29^ in the community.

This review was conducted as the first stage of an intervention development process for people with polypharmacy aged 65 years and over who are not living with dementia or cognitive impairment. It was therefore necessary to exclude papers focussing on people with dementia. Also, a systematic review of evidence assessing interventions to support medicines management in this population has recently been published.^29^

### 2.2 Review process

A team of 10 researchers conducted the review. For consistency, one researcher (G. P.) was involved in all the stages. Title screening was conducted by one reviewer only (G. P.), adopting a conservative approach, and at least a third of the excluded papers were screened by another reviewer^32^ (either R. C. or R. H.). Abstract screening was conducted by eight different researchers (G. P., B. F., V. C., D. A., D. O., C. P., J. S., G. W. P.); references were split among them, who all used the same screening checklist. Before that, each item in the checklist was discussed with the team in a meeting, using practical examples to reach a shared interpretation. At least 50% of abstracts were screened by a second reviewer (either R. C. or R. H.), to ensure consistency and avoid bias.

A screening checklist was designed for full‐text screening and differed from the abstract checklist, with the second being more restrictive. Exclusion criteria relative to type of publication (no protocol study paper, no conference abstract) were added. G. P. and B. F. tested the checklist on five articles. After team discussion, the checklist template was improved and then used by all reviewers involved in full‐text screening (G. P., B. F., V. L., D. O., G. W. P.). At least 50% of papers were screened for full text by more than one reviewer.

Rayyan®, a web app to conduct collaborative literature reviews online,^36^ supported the work of the team. Both during abstract screening and during full‐text screening, disagreements were resolved via discussion. On Rayyan®, disagreements between researchers become visible in real time. This allowed G. P. to promptly address them and facilitate discussion between researchers. Resolved disagreements were also discussed as examples at team meetings to improve consistency. If researchers suspended their judgement, the final decision was made by BF, the principal investigator of the funded study of which this review is part.^29^

A table was created by G. P. and B. F. in Microsoft Excel to extract the data. G. P. identified and selected information relevant to the research question to be extracted and rephrased. The form included author (s), year of publication, country, aims of intervention, study design, length, participants, outcomes and measures, description of intervention, aspects of medicine self‐management addressed and delivery. The form was tested on 20 papers by G. P. and then refined after discussion with B. F. Subsequently, additional columns were added: ‘type of intervention’ (e.g., medicines review and coaching) and ‘effect of intervention’ and the column ‘delivery’ was split into two (‘where’ and ‘by whom’). Data extraction was conducted by G. P. and assessed for accuracy by B. F. Data were also extracted about patient capabilities that the intervention sought to enhance, based on Hollnagel's Safety II resilient abilities: the ability to monitor: to respond; to anticipate; and to learn.^37^

### 2.3 Data synthesis

Heterogeneity of data sources (e.g., different study design, different outcome measures) as well as the aim and the time scale of this review made quality appraisal and statistical meta‐analysis not suitable or necessary.^32^, ^38^ Where feasible, indication of the outcome measures and the effect of the intervention were reported (Table 2). A narrative synthesis approach was applied to summarize the interventions identified and their components (e.g., grouping together studies with similar characteristics, categorizing, describing outcomes and context)^32^ and to identify gaps. Data synthesis was undertaken by G. P. reviewed by B. F. for completeness. B. F. and J. T. independently checked a subset of articles where aspects of the intervention addressed one or more resilient abilities.^37^

## 3 RESULTS

A to tal of 14,353 records were identified and 14,320 were excluded.

**Figure 1:**
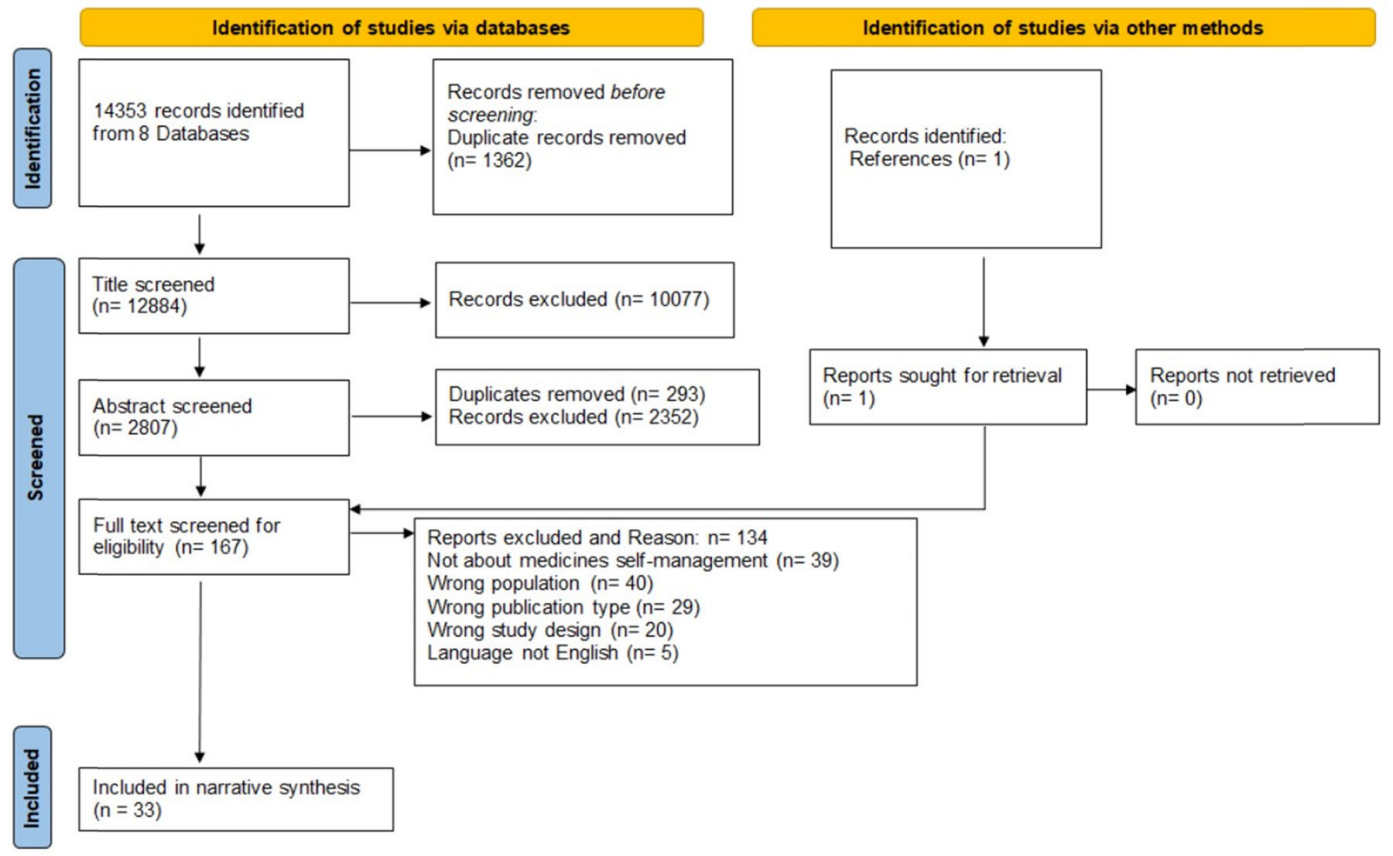
PRISMA Preferred Reporting Items for Systematic Reviews and Meta‐Analyses Diagram.^36^

The process followed for the screening is reported in the PRISMA diagram (Figure 1).

Thirty‐three studies were included in the review (listed in Table 1). Two articles^39^, ^40^ are about the same international trial; however, the first^40^ only presents the results from Northern Ireland (United Kingdom) and was found through database search, and the second^39^ presents the overall results from seven countries and was found cited in the first paper (Figure 1, PRISMA diagram). The rational to include an additional article that was not found through database searches was to provide a better understanding of the intervention. Details related to geographic area of intervention, intervention type, sample and setting characteristics are reported in Table 1.

**Table 1:** Authors and year, country, eligibility, sample, type of intervention, delivered by and where.

Authors, year	Country	Eligibility	Sample size	Type of intervention	Delivered by	Delivered where
Bernsten, C. et al., 2001^39^	Denmark Germany Netherlands Northern Ireland Portugal Republic of Ireland Sweden	Community‐dwelling pharmacy clients aged 65+ taking 4 or more prescribed medicines.	*n* = 2454. Intervention group *n* = 1290, Control group: n = 1164. Mean age 74 (SD = 8). Patients assessed at baseline = 2454, patients assessed at 6 months *n* = 1977, at 12 months *n* = 1627, at 18 months *n* = 1340.	One‐to‐one structured medicines review + education	Pharmacists	Both in community pharmacies and people's homes
Sturgess, I. K. et al., 2003^40^	Northern Ireland	Community‐dwelling pharmacy clients aged 65+ taking 4 or more prescribed medicines	*n* = 191. In intervention: *n* = 110, mean age: 73.1 (SD = 5.0), in control group: n = 81, mean age 74.2 (SD = 6.3). *n* = 147 at 6 months assessment (86, 61); 119 at 12 months (76, 43) and 110 at 18 months (75, 35)	One‐to‐one structured medicines review + education	Pharmacists	Both in community pharmacies and people's homes
Akers, J. L. et al., 2019^41^	United States of America	Low‐income housing residents aged 65+	*n* = 16. Mean age 78, age range: 68–97 years.	One‐to‐one medicines review + education	Pharmacists	At people's homes.
Benoit M. L., 2016^42^	United States of America	Community‐dwelling 65+ adults and carers with chronic conditions.	*n* = 31. Age range 65–80 years. Completed data available on 25 patients only.	Group education + optional medicines review	Nurse and pharmacist	At older adults’ centres.
Bilotta, C. et al., 2011^43^	Italy	Community‐dwelling 65+ adults who had a recent change in medicines or their informal carers	*n* = 120 recruited. *n* = 108 (54 control, 54 intervention group) were tested at the 1‐month follow‐up. Mean age = 80, range 65–95.	One‐to‐one dictated medicine instructions.	Geriatricians	At the geriatric ward
Fulmer, T. et al., 1999^44^	United States of America	community‐dwelling congestive heart failure patients, aged 65 + , prescribed with an ACE inhibitor, calcium channel blocker or beta‐blockers	*n* = 60 recruited, *n* = 50 included in the analysis. The mean age was 74.2 years (SD = 6.8).	One‐to‐one call reminders.	Research team	On the phone
Griffiths, R. et al., 2004^45^	Australia	patients aged 65+ taking oral medicines and receiving community nursing visits and/or informal carers	*n* = 111 assessed for medicine management skills. *n* = 48 eligible to join a nurse‐led intervention. *n* = 24 joined and completed the intervention, alone or with an informal carer. Mean age = 76.7 years (SD = 6.1)	One‐to‐one medicines review + education.	Nurses	At people's homes
Hayes, T. L. et al., 2009^46^	United States of America	Community‐dwelling, living alone, identified as ‘poor adherents’ in a previous study	n = 10. Mean age 82.7 years (SD = 6.4). Complete data only on 7 patients.	Medication monitoring and reminding system	Research team	At people's homes
Holden, R. J. et al., 2020^47^	United States of America	Older adults, aged 60+, having been prescribed with an anticholinergic	*n* = 23 took the App usability test. *n* = 17 (subset) did a feasibility test. The mean age was 67.6 years (SD = 7.8) range 60–85. Not all reported age.	Educational App.	Research team	At the GP clinic's waiting room.
Insel, K. C., Cole, K. L., 2005^48^	United States of America	Community‐dwelling older adults self‐administering one (or more) prescription medication	*n* = 27. Mean age = 78 years, age range 67–89 years.	One‐to‐one tailored education	Nurses	At people's homes.
Insel K. C., et al., 2016^49^	United States of America	Community‐dwelling adults aged 65+ self‐managing an antihypertensive medicine	*n* = 128. *n* = 116 (58 in each group) completed and were included in the analysis. Mean age 77.0 (SD = 7.4)	One‐to‐one tailored education	Nurses	At people's homes.
Lagerin, A. et al., 2014^50^	Sweden	Community‐dwelling older adults aged 75 years.	*n* = 113. Mean age 75 years (SD = 0)	One‐to‐one assessment and education	Nurses	At people's homes
LeBlanc, R. G., Choi, J. 2015^51^	United States of America	Community‐dwelling older adults aged 65+	*n* = 25, mean age 80 years, SD = 10.1. Medicine self‐management measures were included in the analysis for *n* = 17 participants only.	One‐to‐one assessment and tailored	Nurses	At people's homes
Lenaghan, E. et al., 2007^52^	United Kingdom	Community‐dwelling adults aged 80+ prescribed with 4+ oral daily medicines	*n* = 136 recruited and randomized either in the intervention (*n* = 69) or the control group (*n* = 67). Primary outcomes measured for all. Secondary outcomes were measured for *n* = 56 in the intervention group and *n* = 49 in the control group.	One‐to‐one medicines review + education	Pharmacist	At people's homes
Martin, D. et al., 2012^53^	United States of America	Community‐dwelling older adults attending a community centre or their informal carers	*N* = 20. Mean age 75.3 years (SD = 8.8), age range 59–89 years. 1 informal carer joined the pilot.	Medicine reconciliation + tailored pictorial instructions + teach back education	Pharmacist	Not specified
Martin, B. A. et al., 2016 ^54^	United States of America	Community‐dwelling older adults attending Aging and Disability Resource Centres.	*n* = 185 completed both programme sessions; *n* = 145 were included in the final analysis. Mean age 71.5 years (SD = 10.5). 77% were aged 65 years and older.	Group education delivered	Trained lay leaders	At Ageing and Disability centres
Meyer, M. et al., 2021^55^	United States of America	Older adults aged 55+, at risk of ‘unsafe medication management’ (i.e., having 3+ conditions, taking 5+ medicines) or their informal caregivers.	*n* = 180 community‐dwelling adults (patients) and *n* = 77 informal caregivers of patients recruited with Medi‐Cog < 8. The mean age of the patients was 74.4 years (SD = 8.6). *n* = 105 patients and *n* = 35 caregivers completed the intervention.	One‐to‐one medicines review + education.	Pharmacist and nurse	At people's homes and on the phone
Miller, M. J. et al., 2008^56^	United States of America	Older adults attending older adults' centre.	*n* = 118. The mean age was 75.01 years (SD = 7.5) years. *n* = 106 participants were included in the final analytical sample.	Group education delivered by pharmacists at seniors’ centre.	Pharmacists	At older adults’ centres
Murray, M. et al., 1993^57^	United States of America	Public housing older residents, aged 60+, taking 3 or more medicines	*n* = 36 recruited. *n* = 31 randomized and included in the analytical sample. Control Group, *n* = 12, mean age 71.3 years (SD = 5.5) Intervention group 2, *n* = 10, mean age 72.5 years (SD = 10.1). Intervention group 3, *n* = 9, mean age 72.9 years (SD = 6.1).	Adapted packaging+ simplified dosing timing + follow‐up home visits	Pharmacists	At people's homes
Neafsey, P. J. et al., 2001^58^	United States of America	Community‐dwelling older adults taking calcium supplements, antacid or H2 blockers	*n* = 60. The mean age in the intervention group was 68.8 years (SD = 11.9). The mean age in the control group was 73 years (SD = 7.0).	Educational software on PC	Research team	At University
Park E, Kim J. 2016^59^	South Korea	Community‐dwelling adults aged 65+, living in poverty, with hypertension, uncontrolled blood pressure or their informal carers	*n* = 13,452 patients completed the intervention. Number of caregivers not reported. 37.0% of patients were aged 65–74 years and 63.0% were aged 75 years or older.	One‐to‐one education. Delivered by nurses at people's home.	Nurses	At people's homes
Park, M., 2011^60^	South Korea	Community‐dwelling older adults aged 65+ attending senior centre	*n* = 150 older adults from senior centres, aged 65+. *n* = 136 completed the intervention, 46 in group 1, 45 in group 2 and 45 in group 3. The mean age of the 136 patients who completed the intervention was 76.0 years (SD = 6.0).	Group pictorial education session or pictorial booklet	Research team	At older adults’ centres
Parker, R. et al., 2012^61^	New Zealand	Community‐dwelling older adults aged 70+ taking at least 3 medications daily	*n* = 31 were recruited and completed the intervention.	Medication reminder set‐up and monitoring service.	GP, pharmacist, research team	At people's homes
Poureslami, I. et al., 2016^62^	Canada	Older migrants, Mandarin or Cantonese speakers diagnosed with COPD, who had lived in Canada for 20+ years	*n* = 91 were randomized into four groups (Group A: *n* = 22, Group B: *n* = 26, Group C: *n* = 29, Group D (control): *n* = 14) and completed posttest measures. The median age was 75 years, SD not reported. 59,3% of the participants were aged 75 years or older.	Co‐produced educational videos and pamphlet.	Research team	At the COPD clinic
Schulz, R. M. et al., 2011^63^	United States of America	Community‐dwelling Medicare frail older waivers, clients of 12 pharmacies with at least one active prescription.	*n* = 273 patients were matched with 800 other Medicare clients with similar characteristics (control group). The mean age was 71.95 years (SD = 15.1).	Tailored calendar card with medicines and care coordination.	Pharmacist and health educator	At people's homes
Sidel, V. et al., 1990^64^	United States of America	Community‐dwelling adults aged 65+ assessed as ‘high risk’.	*n* = 256 recruited. Intervention group n = 113, 65–74 = 48.4%, 75–84 = 38.5%, 85 and over = 13.2%. Control group, *n* = 143, 65–74 = 48.1%, 75–84 = 41.4%, 85 and over 10.6%. *n* = 92 in the intervention group and *n* = 104 in the control group were re‐assessed postintervention	One‐to‐one education.	Pharmacist	At people's homes
Suzuki, R. et al., 2018^65^	Japan	Community dwelling elderly, on one or more prescribed medicines, who reported difficulties in remembering to take their medicines as instructed, and their informal carers.	*n* = 30, 10 elderly patients and 20 medication supporters (carers) of patients receiving a one‐dose package. Patient's mean age was 80.3 years (SD = 6.6).	One dosage pouched medicine administration system	Research team	At people's homes
Wang, C. J. et al., 2013^66^	Taiwan	Community‐dwelling adults aged 65+, living in rural areas and diagnosed with 2+ chronic illness	*n* = 62 were recruited and included in the analytical sample, 32 in intervention and 30 in routine care. The mean age of the sample was 71.3 years (SD = 7.8).	One‐to‐one education and coaching	Trained lay leaders (community volunteers)	At people's homes
Whittaker, C. F. et al., 2017^67^	United States of America	Community‐dwelling older adults attending senior centres	*n* = 101 recruited, *n* = 53 completed the intervention, *n* = 35 at 30 days of follow‐up. In the game group (*n* = 27), 23 were 70 years of age or older and 25 were 60+; in the brochure group (*n* = 26), 16 patients were over 70 years of age and 25 patients were 60+.	Group educational interactive game, facilitated by pharmacists at seniors’ centres.	Pharmacists	At older adults’ centres
Wong, A. K. C. et al., 2019^68^	Honk Hong	Community‐dwelling older adults aged 60+, mobile, with no advanced cognitive impairments	*n* = 457 randomized either in the intervention (*n* = 230) or the control group (*n* = 227). The mean age was 78 years (SD = 7.92). 19 patients in the intervention and 9 patients in the control group did not complete the intervention, but were included in the analysis.	One‐to‐one education. Home visits and follow‐up calls delivered by nurses and community workers.	Nurses and community workers	At people's homes and on the phone
Poonprapai P. et al., 2022^69^	Thailand	Community‐dwelling older diabetes patients, aged 65+, with poor condition management and their family carers	*n* = 166 dyads were recruited and randomized; *n* = 157 completed the intervention, 78 in the intervention group and 79 in the control group. The mean age of patients in the intervention group was 67.36 years (SD = 5.72); the mean age of patients in the control group was 67.80 years (SD = 6.18).	Educational App sharing daily Infographics with family carers	Research team	On the phone (App)
Zhang, S. et al., 2022^70^	China	Community‐dwelling older patients, aged 65+, with 1+ chronic condition, having taken 5+ medicines for 3 months or more	*n* = 448 patients joined the study; *n* = 412 remained until the end and were included in the analysis. Mean age = 73.43 years (SD = 7.8).	One‐to‐one medicines review + education delivered by pharmacists at people's homes.	Pharmacist	At people's homes
Falamić, S. et al., 2021^71^	Croatia	Community‐dwelling older patients, aged 65+ patients, living in rural areas and prescribed Warfarin for 3+ months.	140 people joined the study and were randomized into two groups of 70 each. 131 participants finished the study and were included in the analysis. The median age was 73 years (IQR: 70–80).	One‐to‐one warfarin therapy review + education.	Pharmacist	Not specified

Abbreviations: IQR, interquartile range; Medi‐Cog, medicines cognition and ability to safely manage medicines.

### 3.1 Country of intervention

The majority of studies (*n* = 17) were conducted in the United States, two in the United Kingdom (one in England and one in Northern Ireland), two in South Korea and one each in Sweden, Japan, Australia, New Zealand, Hong Kong, Italy, Canada, China, Thailand, Croatia and Taiwan. One intervention^39^, ^40^ was developed and implemented by an international network of seven European countries.

### 3.2 Study design

Most articles described the study design as pilots (*n* = 9) or randomized‐controlled trials (RCTs) (*n* = 11). Most randomized‐controlled studies had two arms,^39^, ^49^, ^52^, ^64^, ^68^, ^69^, ^71^ two had three arms^44^, ^57^ and one had four arms.^62^ Other designs included:

- Quasi‐experimental design with two^67^ or three arms.^60^
- Single group with a pretest posttest evaluation design.^46^, ^51^, ^54^, ^59^
- Single group with a pretest posttest evaluation with a cross‐sectional survey design.^45^
- Modified, separate‐sample, pretest posttest study.^56^
- Retrospective analysis of patient care documentation.^41^
- Exploratory study of unsafe medication management.^50^
- Evaluation of the medicine support system.^65^
- Prospective cohort studies.^63^, ^70^

### 3.3 Target and population

The intervention was offered to informal carers/medicines supporters as well or on behalf of patients in only 7 out of 33 articles. Where reported (in 26 out of 33 articles), the mean age of the participants varied from 67.8 years (SD = 7.8)^47^ to 82.7 (SD = 6.4).^46^ In a minority of studies, the intervention explicitly targeted patients taking multiple medicines^39^, ^52^, ^55^, ^57^, ^61^, ^70^ or living with multiple chronic conditions.^42^, ^55^, ^66^ Some interventions targeted people with specific conditions such as congestive heart failure,^44^ hypertension,^49^, ^59^ diabetes^69^ and COPD^62^ or taking specific types of medicines, e.g., anticholinergic,^47^ anticoagulants^71^ or antacids.^58^ Only one of the studies identified explicitly targeted older people living with frailty.^63^

### 3.4 Intervention types

The most common intervention component delivered was education about medicines (*n* = 26), alone or combined with other components. In eight articles, education was delivered in conjunction with a medicines review.^39^, ^40^, ^41^, ^45^, ^52^, ^55^, ^70^, ^71^ In five studies,^47^, ^50^, ^51^, ^68^, ^69^ education followed assessment of anticholinergic risk,^47^ safe management of medicines,^50^ ability to manage medicines,^51^ diabetes self‐care abilities^69^ and comprehensive assessment.^68^ Education was delivered both one to one^39^, ^40^, ^41^, ^45^, ^48^, ^49^, ^50^, ^51^, ^52^, ^53^, ^55^, ^59^, ^60^, ^64^, ^66^, ^68^, ^70^, ^71^ and in groups.^42^, ^54^, ^56^, ^60^, ^67^ Techniques and tools included coaching,^41^, ^48^, ^49^, ^66^ role play,^54^ teach‐back/show‐back,^53^, ^60^, ^66^ group discussion,^58^, ^60^ dilemma scenarios,^58^, ^60^ pictorial information or visual maps,^53^, ^60^, ^62^, ^66^ video tutorials,^62^ apps and software.^47^, ^58^, ^69^ In only three interventions^58^, ^60^, ^62^ was the educational material codesigned or pretested by either patients or relevant stakeholders.

Other types of interventions included new way to communicate administration instructions by HCPs with^53^ or without additional education,^43^ new dosage packaging systems^57^, ^63^ and new ways to remind patients to take their medicines,^44^, ^46^, ^61^, ^65^ such as call reminders, preprogrammed alarms and visual reminders, in some cases part of automatic dispensing systems installed in the patient's home.^46^, ^61^, ^65^

### 3.5 Who delivered the intervention and where

The majority of interventions (*n* = 20) were delivered in people's homes. A range of HCPs, including pharmacists, pharmacy technicians, nurses, GPs, consultants, researchers, social workers and community workers, were involved. Most interventions were delivered by either a pharmacist,^39^, ^52^, ^53^, ^56^, ^57^, ^64^, ^67^, ^70^, ^71^ a nurse^45^, ^48^, ^49^, ^50^, ^51^, ^59^ or a team of two or more HCPs.^42^, ^55^, ^61^, ^63^, ^68^ In two articles,^54^, ^66^ the intervention was led by trained lay people recruited in the community.

Details related to study design, intervention length, aims and outcomes of intervention, aspects of medicines management addressed and indication effect of intervention are reported in Table 2.

**Table 2:** Author, year, study design, intervention length, measures, description of intervention, aspects of medicine management addressed, aims, outcomes and effect of intervention.

Authors, year	Study design	Intervention length	Measures	Description of intervent Knowledge of medicineion	Aspects of medicine management addressed (and techniques adopted)	Aims	Outcomes	Effect of intervention
Bernsten C. et al., 2001^39^	2‐arm longitudinal RCT with repeated measures	18‐month programme. Participants assessed at baseline, and at 6, 12 and 18 months	Knowledge of medicines and adherence (self‐reported): ad hoc questionnaires. Change in medicines: self‐reported. Quality of Life: 36‐Item Short Form Health Survey (SF‐36).	Intervention group participants received medicines' review + education programme including customized education/advice and individual plan (e.g., simplified regiments) by a pharmacist at home. The control group received care as usual.	Adherence Storage of medicines Knowledge of medicines	Increase adherence.	A significantly higher ratio of patients in the intervention group changed from scoring noncompliant to compliant, compared with patients in the control group (15.2% vs. 12.2%, *χ* ^2^, *p* = .028) at 18 months. Quality‐of‐Life measures declined over time in both groups. Differences in knowledge of medicines, self‐reported change in medicines, hospitalizations and the average overall cost of care per patient were not significant. Not all outcome measures were available for all the countries involved.	At 18 months, the ratio of patients who scored ‘compliant’ increased by 15.2% in the intervention group, while the increase in the control group was 12.2%.
						Increase knowledge of medicines.		
						Encourage medicines' optimization.		
						Improve patient quality of life.		
					Patients were educated on conditions, medicines and administration techniques. Patients received compliance aids tools (e.g., medicine chart)	Reduce cost of care.		
						Reduce hospital admissions		
Sturgess, I. K. et al., 2003^40^	2‐arm longitudinal RCT	18‐month programme. Participants assessed at baseline and at 6, 12 and 18 months	Knowledge of medicines and adherence (self‐reported): ad hoc questionnaires. Change in medicines: self‐reported. Quality of Life: 36‐Item Short Form Health Survey (SF‐36).	Intervention group participants received medicines review + education programme including customized education/advice and individual plan (e.g., simplified regimens). The control group received care as usual.	Adherence Storage of medicines Knowledge of medicines	Increase adherence.	At 18 months, the percentage of patients scoring as compliant in the intervention group was significantly higher compared with that in the control group (47.3% vs. 14.7%). (*p* < .05). Problems with medicines in patients in the intervention group were significantly less than those in the control group (0.90, SD = 1.27 vs. 2.09 SD = 2.38, *p* < .05) only in the last 6 months. The average cost of care was significantly lower for patients in the intervention group, compared with the patients in the control group. Changes in knowledge of medicines, hospitalizations and health‐related quality of life were not significant.	At 18 months, a significantly higher percentage (47.3% vs. 14.7%) of patients in the intervention group scored ‘compliant’, compared with patients in the control group. Significant differences were also found in the cost of care and problems with medicines.
						Increase knowledge of medicines		
						Encourage medicines' optimization.		
						Increase patient quality of life, Reduce cost of care.		

					Patients were educated on conditions, medicines and administration techniques. Patients received compliance aids tools (e.g., medicine chart)			
						Reduce hospital admissions		
Akers, J. L. et al., 2019^41^	Retrospective analysis of patients' documentations (data from January 2012 to June 2016).	Initial visit and monthly follow‐ups. The total length of the intervention varied for each patient.	Numbers and types of problems with medicines. Number and type of intervention started.	Patients received medicines review + education by a pharmacist at home. Intervention included assessment of problem with medicines, customized education, follow‐up tasks and support for coordination of care.	Education included Knowledge of conditions medicine administration techniques. Patients received compliance aid tools. Techniques included coaching, goal‐setting, shared list of follow‐up tasks.	Increase adherence.	At visits, pharmacists identified 94 problems with medicines, 5.9 problems per patient. The most common problem was nonadherence (36%). 90 interventions were initiated. Education was the most common (34%), followed by discontinuation of medicines (12%).	Not applicable
						Increase knowledge of medicines.		
						Encourage medicines' optimization.		
Benoit, M. L., 2016^42^	Pilot of programme. Single‐group pretest and posttest.	2 h educational session and 2 h hands‐on seminar. Participants were assessed at baseline and after the education session.	Knowledge of medical product safety: National Council on Patient Information and Education ad hoc tool.	Group education + optional medicines review (Brown Bag) delivered by nurses and pharmacist at older adults' centres	Education on adherence and medication safety covered Set up of medicines Storage of medicines Disposal of medicines Keeping a medicine list Communication with healthcare professionals Tools to support adherence (e.g., calendar reminder)	Increase adherence.	Adherence measures were not reported and communication with the healthcare team was not assessed. Pretest results showed limited medical product knowledge (*M* = 28.64, SD = 32.62). The post‐test results indicated a significant increase in medical product safety knowledge after the educational session (M 79.24, SD = 21.32, *t*[24] = −7.96, *p* < .001).	Medical product safety knowledge increased by 50.6 points in post intervention measures (scale range not reported).
						Increase knowledge of medical product safety.		
						Improve communication with healthcare professionals.		
Bilotta, C. et al., 2011^43^	Pilot with 2 randomized groups	Time required to dictate instructions. Participants were assessed at baseline and a month after the intervention.	Errors in adherence, including forgetfulness: self‐reported by patients or informal carers	For patients and carers in the intervention group, the specialist (geriatrician) dictated the medicine list and administration instructions to the patient and/or carers. Patients and carers in the control group received care as usual.	Adherence to administration schedule and instructions. Patients/carers in the intervention group received detailed medicine administration instructions and the opportunity to write them down verbatim, to be able to refer to them at home.	Increase adherence by reducing errors in administration.	In the intervention group, the prevalence of adherence errors decreased from 70% to 20% after 1 month; in the control group, it decreased from 67% to 59%. The intervention group was found to have a lower risk of making errors in the adherence to pharmacological treatment when compared with the control group (odds ratio [OR]: 0.16, 95% confidence interval [CI]: 0.06–0.39; *p* < .001).	In the intervention group, the prevalence of adherence errors decreased by 50% a month after the intervention; in the control group, it decreased by 8%.
Fulmer, T. et al., 1999^44^	3‐arm RCT	6 weeks. Compliance was assessed at baseline (for 2 weeks pre intervention), after 6 weeks and after 8 weeks. Quality of life was assessed pre and post intervention.	Compliance: Medication Event Monitoring System caps. Quality of Life: 36‐Item Short Form Health Survey (SF‐36) and Living with heart failure questionnaire (MLHF).	Participants in intervention groups received either daily phone call (A) or video call (B) reminders for 6 weeks to improve medication compliance to heart failure medicines. The control group received care as usual.	Adherence. Patients in intervention groups received call reminders to take heart failure medicines.	Increase adherence.	Average compliance in the control group decreased significantly from 81% at baseline to 57% at two weeks after the end of the intervention (*p* < .04), but remained stable in both intervention groups (no more than 2% change). No significant difference was found between receiving a call and video call. Changes in Quality of Life were not significant for SF‐36 results, while MLHF results improved significantly (*p* < .001) in all groups, but differences in the increase between the groups were not significant.	In the control group, the average compliance score decreased by 24% in 10 weeks. In both intervention groups, average compliance remained stable.
						Improve patient quality of life.		
Griffiths, R. et al., 2004^45^	Single‐group pretest and post with a cross‐sectional survey.	Varied. Follow‐up after 4 weeks	Adherence: self‐reported or combined with pill count. Ability to manage medicines: Romonko and Pereles technique.^68^ Complexity of the regime: Medication Complexity Index (MCI).	Medicines review and education delivered by a nurse at home. Assessment of adherence, medicine knowledge and ability to manage, followed by customized education.	Tailored education included Knowledge of medicines (function, doses, special instructions and schedule) Use of adherence aid tools Nurse facilitated care coordination: Providing Medication list Talking to a pharmacist and GP to encourage optimization	Increase adherence.	4 weeks after the intervention, no significant difference was found in (1) adherence or nonadherence; (2) total number of medications; and (3) Medication Complexity Index scores. Knowledge of medicines increased significantly for what concerns ‘remembering names of medicines’, with the mean score going from 82.3% (SD = 35.0) to 97.30% (SD=10.5), (*p* < 0.029), and 'remembering scheduling of medications', with mean score going from 86.6% (SD = 30.2) to a 97.90 (SD = 10.2), (*p* < 0.038). Changes in score related to 'knowledge of purpose of medicines' were not significant.	In measures taken after the intervention, participants' score in medicines knowledge increased by 15%, for what concerns 'remembering names of medicines', and by 11.3%, for what concerns ‘remembering scheduling of medicines’.
						Increase knowledge of medicines.		
						Reduce complexity of the medicine regimen.		
Hayes T. L. et al., 2009^46^	Single group with 3‐period repeated measures.	The total length of the intervention varied for each patient.	Adherence measured by the system for 10.7 weeks (SD = 4.4) in the first phase, for 10.1 weeks (SD = 3.3) in the second phase and for 8.4 weeks (SD = 4.2) in the third phase	Medication monitoring and reminding system installed in participants’ homes. Participants were asked to take a vitamin d tablet at the same time. In the first phase, there were no prompts. In the second phase, prompts were sound and visual alarms at the set time. In the third phase, patients were prompted only when the system inferred that they were likely to miss their dose.	Adherence. Participants received different types of reminders by a medication monitoring and reminding system to take a dose of vitamin at a set time.	Increase adherence.	At baseline, the mean adherence rate (95% CIs) was 68.1% [57.5–80.5]. The mean adherence increased up to 73.5% [68.0–78.6] in the time‐based reminder phase and increased to 92.3% [84.7–97.0] in the context‐based reminder phase.	Measures of mean adherence increased by 5.4% during the timed alarm reminder phase and by 24.1% during the context‐based reminder phase.
Holden, R. J. et al., 2020^47^	Single‐group usability and feasibility pilot.	Patients tested an app waiting to see a physician and were interviewed 24 h after their appointment.	Usability and feasibility of app. Number of conversations about anticholinergic risk started.	Patients tested an android app to measure anticholinergic risk and empowered them to raise their concerns about the risk at medical appointments. Patients who scored as ‘high risk’ received a brochure and were advised to start a conversation about anticholinergic risk with their doctor	Knowledge of medicines: anticholinergic risk. Communication with healthcare professionals: raising concerns about medicines	Increase knowledge of anticholinergics.	Brain Buddy was found to have acceptable usability. All participants who took part in the feasibility test reported that after using Brain Buddy, they felt better informed about anticholinergic risks. 82% of the participants (11 out of 17) did speak to their physician about anticholinergic risks.	No outcome measures reported for knowledge of medicines.
						Provoke conversations with HCP about risks associated with anticholinergic medicines.		
Insel, K. C., Cole, K. L., 2005^48^	Pilot of programme with single‐group pretest and posttest.	Not specified Adherence electronically measured for 8 weeks before and after the intervention.	Electronically measured adherence (percentage of days when the correct number of doses was taken).	Tailored education to enhance adherence and adherence monitoring delivered by a nurse at home.	Participants were coached to: establish routines to remember to take medicines enhance adherence using visual clues (e.g., keeping medicines visible) monitor if medicines were taken as intended (e.g., using a pill box).	Improve adherence.	The percentage of days when patients took the correct number of doses increased from a mean of 64.5% at baseline to 78% after the intervention (Z score on the Wilcoxon signed ranks test = 2.5, *p* < .01).	Percentage of days when correct doses were taken increased by 13.5% in the 8 weeks after the intervention.
Insel, K. C., et al., 2016^49^	2‐arm RCT.	Once a week visit for 4 weeks. Measures were taken at baseline and at 4 weeks and after 5 months.	Adherence was measured electronically and calculated as the percentage of adherence in interval doses.	All participants were visited by a nurse once a week for 4 weeks and received information about the antihypertensive condition and medications. In addition, participants in the intervention group received tailored education.	Participants in the intervention group were coached to establish routines to support remembering using automatic associative processes, set up and use medication organizer monitor if medicines were taken as intended.	Improve adherence.	In the intervention group, the mean adherence improved significantly after 4 weeks, from 57.41% (SD = 29.84) at baseline to 77.78%, (SD = 24.42), *p* < .001), but the increase was not sustained over the five‐month monitoring period (59.0 SD = 32.7, *p* < .001). Adherence in the control group declined slightly in the first 4 weeks and significantly from week 4 to month 5 (from *M* = 67.8 SD = 28.5 at baseline to *M* = 61.1 SD = 29.9, *p* = .01 at 5 months). The use of newly introduced strategies significantly declined after 5 months (from a mean of 10.9 to a mean of 8.8 (*t*(1,57) = 7.70, *p* < .001).	In the intervention group, the mean adherence score increased by 20.37% at week 4, but then decreased dramatically to a 1.59% increase 5 months after the intervention. In the control group, the mean adherence score decreased during the time of the study.
Lagerin, A. et al., 2014^50^	Exploratory study	Intervention length varied for each participant	Safe use of medicines assessed with the Safe Medication Assessment tool (SMA)^69^	Patients receiving their routine 75 years visit, part of the Swedish healthcare system, were assessed for safe use of medicines. Patients who scored as ‘at risk’ received tailored information and education, and link with other health and social care professionals was facilitated.	Tailored education included establishing routines to remember to take medicines knowledge of medicines' indication medicine storage alcohol and meds interactions.	Improve knowledge of medicines.	The median score in the assessment the Safe Medication Assessment tool was 25 (range 9‐28), and 42.5% of the participants scored higher than 25. The use of five or more drugs was the most common factor in patients who scored as ‘high risk’. 81 out of 113 participants received nursing care interventions (71.7%) after the assessment; in most cases, it consisted of information and education.	Not applicable.
						Assess risks in medicine management and alert HCP.		
LeBlanc, R. G., Choi, J. 2015^51^	Single‐group pretest and posttest.	Participants were assessed before the intervention and reassessed 1–2 weeks after the intervention.	Ability to self‐manage medicines assessed with the Drug Regimen Unassisted Grading Scale (DRUGS)^70^	Assessment of understanding of medicines and ability to manage regimen (e.g., identify medicines, open containers, get the correct dose at the right time) + tailored education and follow‐ups were delivered at home by a nurse. In follow‐up visits, the nurse assessed (a) if medicines were optimized by prescribers, (b) if the list provided was used by patients and professionals and (c) reinforced advice.	Tailored education included knowledge of medicines medicine administration Nurse also facilitated coordination of care: reporting medicine‐related problems to primary care; sharing the updated medicine list with the care team.	Improve ability to identify, access, dose and time medicines.	Changes in patients' ability to identify, access, dose and time medicines were not significant. The percentage of participants using a medication list increased from 48% to 90% after the intervention. Of the 68% of participants found to have medicine‐related problems, only 8% had an optimization change in their therapy during the time of the project.	The percentage of patients using a medicine list increased by 42% after the intervention.
						Encourage medicines' optimization		
						Encourage change in behaviour: use of a medicine list		
Lenaghan, E. et al., 2007^52^	2‐arm RCT	Participants were assessed at baseline at the follow‐up visit (at 6–8 weeks)		Patients in the intervention group received medicines review and education delivered by a pharmacist at home; patients in the control group received care as usual. After assessing problems with medicines and storage issues, the pharmacist provided patients in the intervention group with tailored education.	Education included tailored advice compliance aid tools Pharmacist also facilitated coordination of care contacting GP regarding issues making changes in the medicine regimen if needed.	Reduce the number of medicines prescribed.	At reassessment, no significant changes were found in the intervention group for nonelective hospital admissions, deaths, care home admissions or quality of life. In the control group, the mean number of items prescribed increased from 9.85 to 10.33; in the intervention group, the mean number decreased from 9.01 to 8.68. The decrease in the number of medicines prescribed to the intervention group was significant. The difference in change between the two groups was significant (−0.87 items in favour of the intervention group, 95% CI: −1.66 to −0.08, *p* = .03).	No significant changes were found in hospital and care home admissions, number of deaths or quality of life. The only significant change was a decrease in the mean number of prescribed medicines in the intervention group compared with the control group.
						Reduce hospital and care home admissions.		
						Reduce deaths.		
						Improve quality of life		
Martin, D. et al., 2012^53^	Pilot project with a single‐group pretest and posttest design	Participants used pictorial instructions for 6 weeks. Participants were assessed for adherence and self‐efficacy pre‐ and postintervention.	Adherence was measured with adapted ARMS^71^ (10–40, the higher the worse) Self‐efficacy was measured with SEAMS^72^ (13–39, the higher the greater).	Pharmacist provided illustrated medicine instructions (PictureRx cards) and an educational session.	Participants received tailored pictorial information about medicines' purpose, schedule and administration instructions teach me back session on how to use pictorial instructions	Increase adherence.	After 6 weeks of PictureRx card use, both participants’ self‐reported adherence and self‐efficacy scored improved significantly. The adherence mean score went from 13.3 (SD = 3.2) at pretest to 11.1 (SD = 3.1), (*p* = .046) posttest. Mean self‐efficacy increased from 28.4 (SD = 9.1) to 35.8 (SD = 5.8), *p* < .001). 100% of participants stated that the PictureRx cards were very helpful. Many participants asked to use PictureRX cards after the end of the pilot.	Both adherence and self‐efficacy scores increased significantly in postintervention measurements. Mean adherence improved by 2.2 points in the ARMS scale. Mean self‐efficacy improved by 7.4 points in the SEAMS scale.
						Improve self‐efficacy for taking medicines correctly		
Martin, B. A. et al., 2016^54^	Single‐group pretest and posttest design.	2 h programme. Pharmacist's role knowledge was assessed pre and post intervention. Changes in behaviour and self‐efficacy in communication were assessed pre‐ and postintervention and at 3 months.	Knowledge of the pharmacist's role, self‐efficacy in communication pre intervention and change in behaviour were assessed with ad hoc designed questionnaires.	Educational and behaviour change programme aimed to improve medication communication skills and self‐efficacy in communication with the pharmacist. The programme was delivered by a trained lay leader in a small group at older adult centres.	Content covered by the programme included role of pharmacists; use of medicine list in interactions with a pharmacist preparing and using a list of questions to ask the pharmacist. Learning techniques included brainstorming, role‐play and problem solving.	Improve knowledge of pharmacist role.	Knowledge of pharmacist role measures increased significantly after the programme for 5 out 6 items. Self‐efficacy in communication with pharmacist increased significantly from baseline, at both points in time, for 16 out of 17 items. In terms of behaviour change, 29.2% of participants received a comprehensive medication review and an additional 30% planned to request one. 28.5% of the participants said that they addressed medication schedule issues with the pharmacist.	After the programme, both scores of knowledge of pharmacist role and scores for self‐efficacy in communication with the pharmacist increased significantly from baseline for most items. At 3 months, more than a quarter of the participants had or planned to have a medicines review with a pharmacist.
						Improve self‐efficacy in communication with the pharmacist.		
						Improve quality of interactions/communication with pharmacist.		
Meyer, M. et al., 2021^55^	Single‐group pretest and posttest pilot	The mean length of the intervention was 232 days. Self‐efficacy and adherence were measured before and after the intervention.	Self‐efficacy was measured with the MUSE scale^73^ (8–32, the higher the greater). Adherence with the MedAdhI scale^74^ (0–10, the lower the better). For some patients (diabetes, cholesterol, hypertension), adherence was assessed with pill count too. If patients were unable to manage, carers were assessed	Pharmacy technician or health workers assessed medication‐related problems (e.g., adverse reactions and interactions, poor medicines knowledge, poor adherence, dexterity or visual issues, high burden) at first home visit. Prescribers were notified of problems. Patients received a written medication action plan. A nurse provided tailored education at follow‐up visits and calls.	Education provided by nurses included Use of compliance aids (e.g., how to fill a pill box) Medicines' log How to perform self‐monitoring (e.g., blood sugars and blood pressure) Discarding of medications Administration of medicines (e.g., use of inhaler).	Increase adherence.	Patients’ self‐efficacy mean score increased significantly (*p* < .001) from 24.3 (SD = 4.8) to 28.9 (SD = 4.9) after the intervention. Carers’ self‐efficacy mean score increased from 29.2 (SD = 2.9) at enrolment to 31.3 (SD = 1.3) at the end of the programme (*t* = −5.2, *p* < .001). The mean score in adherence declined significantly (*t* = 9.5, *p* < .001), with the mean MedAdhIR score decreasing from 4.91 (SD = 3.0) to 1.8 (SD = 2.1), indicating significant improvement. Adherence to selected medicines (diabetes, cholesterol, hypertension) measured with pill count also increased from 34.7% (SD = 0.29) to 75.8% (SD = 0.24) by the end of the programme (*t* = −14.58, *p* < .001).	The mean patients’ self‐efficacy score increased by 4.6 points (MUSE scale range: 8–32) after the intervention. The mean carers’ self‐efficacy score increased by 2.1. Patients’ mean adherence score improved by 0.9 points. (MedAdhAIR scale range 0–10), while adherence to diabetes, cholesterol and hypertension increased by 41% at the end of the programme.
						Increase self‐efficacy in managing medicines.		
Miller, M. J. et al., 2008^56^	Modified separate sample pretest and posttest, with random allocation.	45 min presentation. Participants were assessed for readiness to adopt the clear communication behaviour either pretest and posttest (group 1), pretest only (group 2) or posttest only (group 3).	Readiness to adopt clear health communication principles was assessed with ad hoc designed questionnaires	Programme to improve communication with the pharmacist based on clear health communication principles with the pharmacist delivered by pharmacists at older adults’ centres.	Behaviour change addressed by the programme: maintaining a personal complete list of medications sharing the list with the pharmacist maintaining a list of concerns/questions to discuss with the pharmacist Asking key questions when medicines are started (why do I need it? what if I do not take it) inviting friends or family for support at appointments	Encourage patients to adopt clear health communication principles with pharmacists.	In group 1, a significantly higher proportion of participants (from 53.1% in pretest to 62.5% in posttest, *p* ≤ .025) stated that they were carrying or planning to carry a list of medications when going to the pharmacy after the intervention. Changes were not statistically significant for other behaviours. When group 2 (pretest only) and group 3 (posttest only) were compared, a higher proportion of patients intended to involve family and friends in appointments (70.6% vs. 17.6% *p* < .001) and carried or intended to carry a list of questions (81.8% vs. 50%, *p* = .010) and a list of medicines (85.3% vs. 48.5% *p* = .002) in group 3 that was assessed after the programme (group 3).	After the programme, 9.4% more participants in group 1 started to carry/intended to carry a full list of medicines. In addition, in group 3, a significantly higher proportion of patients intended to involve family/friends in appointments (*p* < .001) and planned to bring a list of problems (*p* = .010) and a list of medicines (*p* = .002) to the pharmacy compared with group 2.
Murray, M. et al., 1993^57^	3‐arm RCT nonblind	6 months. Medication compliance was assessed at month 1, 3, 4, 5 and 6.	Compliance was assessed with pill count and open ended questions.	Adapted packaging and timing of dose intervention delivered by the pharmacist. Patients in group 2 used conventional packaging and a simplified twice a day dose timing, while patients in group 3 received specially designed packaging containing all medications to be taken twice a day. Patients in group 1 received standard care.	Medicine calendar card to support adherence. At the monthly follow‐up, pharmacists addressed drug reactions and problems with medications' packaging.	Increase adherence.	The mean compliance score adjusted for study visit (months 1–6) was significantly higher in group 3 compared with groups 1 and 2 (*p* = .017). The mean compliance score was 92.6% (SE = 2.1, range 62.3‐100) in group 3, 82.6% (SD = 2.0, range 4.59‐100) in group 2 and 79.0% (SE = 2.1, range: 14.4–100) in group 1. Compliance did not differ significantly between the control group and the group with simplified timing only.	The group with simplified timing and specially designed packaging (group 3) had a mean compliance score that was 10% higher than that of the group with simplified therapy and 13.6% higher than that of the control group.
Neafsey, P. J. et al., 2001^58^	Pilot test with 2 randomized groups.	Time required to complete computer programme not reported. Measures were taken posttest only.	Knowledge of antacid medicine interactions and self‐efficacy in avoiding interactions were measured with ad hoc designed questionnaires.	Participants in the intervention group joined an interactive programme on a computer to learn about risks of medicine interactions and medicine interactions with alcohol. Participants in the control group received care as usual (no use of software).	Knowledge of medicines: Information about antacid medicines Risk of medicines' interactions when taking antacids Risks of alcohol interactions when taking antacids Techniques included a dilemma scenario with multiple choices and a quiz.	Increase knowledge of antacid medicine–alcohol interactions.	Intervention group participants scored significantly higher in knowledge of potential interactions of medications and alcohol (*M* = 71.7%, SD = 19.1) compared with the ones in the control group (M = 36.2% SD = 16.5, *p* < .001). They also showed greater self‐efficacy (3.14 SD = 0.9 vs. 1.76, SD = 0.99) in avoiding drug and alcohol interaction (*p* < .001, scale range unknown).	After the intervention, the mean score in knowledge of medicine–alcohol interactions was 35.5% higher in the intervention group than in the control group. The mean self‐efficacy score was 1.38 points higher in the intervention group compared with the control group (range not reported).
						increase confidence in avoiding antacid med–alcohol interactions.		
Park, E., Kim, J., 2016^59^	Single‐group pretest and posttest	Programme lasted between 2 and 4 months. Measures were taken at baseline and after the end of the intervention (2–4 months).	Measurement tools for knowledge of condition, adherence (self‐reported) and self‐confidence in condition management were designed by the research team	Education and counselling programme on hypertension, delivered by a nurse at home. Nurse and patient identified shared priorities and goals for condition management and medicine adherence. If needed, patients were referred by the nurse to healthcare professionals and social workers.	Counselling covered self‐management of condition (e.g., keep record of blood pressure, understanding and monitoring symptoms, lifestyle and diet) adherence to hypertension medicines	Increase hypertension medicine adherence.	After the intervention, hypertension knowledge, medication adherence and overall self‐confidence in hypertension management showed significant improvement (all with *p* < .001). Adherence mean score (scale range: 2–10) increased from 4.08 (SD = 1.11) to 4.66 (SD not reported), knowledge of hypertension mean score (scale range: 0–10) increased from 5.17 (SD = 2.36) to 8.38 (SD not reported) and the mean self‐confidence score (scale range: 10–50) increased from 33.03 (SD = 6.12) to 39.26 (SD not reported)	In the post‐intervention assessment, the mean adherence score increased by 0.58 points (2–10), the mean knowledge of hypertension increased by 3.21 points (0–10) and the mean self‐confidence in condition management increased by 6.23 points in the test scale (10–50).
						Increase knowledge of hypertension.		
						Improve self‐confidence in condition management.		
Park, M., 2011^60^	Quasi experimental design with 3 groups nonrandomized pre–post test	40 min sessions were delivered once a week for 3 weeks. Measures were taken at baseline and after the intervention.	Self‐efficacy was measured with the SEAMS^72^ scale. Knowledge of medication safety was measured with a questionnaire designed by the research team	Pictorial group education delivered by the research team at older adults' centres. The first intervention group received an information booklet; the second intervention group received the booklet and education sessions. The control group received standard care (no booklet or education). Booklets and group education were co‐designed by stakeholders and patient representatives.	Content covered by the booklet and group education included guidance on how to read medicine leaflets, calculate medicine dosage, use adherence aids, identify side effects and avoid interactions. Techniques included dilemma scenarios, pictorial cards and maps, group discussion.	Increase knowledge of medication safety.	At the posttest assessment, both intervention groups had significantly higher scores in self‐efficacy and knowledge of medicines safety, compared with the control group, (*p* < .05). The group involved in education sessions scored significantly higher than the group that only read the booklet (*p* < .05). After the intervention, mean self‐efficacy (13–39) increased from 25.33 (SD = 4.06) to 25.36 (SD = 4.01) in the control group, from 26.70 (SD = 3.20) to 28.68 (SD = 3.18) in the booklet group and from 26.30 (SD = 3.92) to 30.61 (SD = 3.36) in the booklet + education group. After the intervention, the mean knowledge score (scale range: 0–15) increased from 8.03 (SD = 1.81) to 8.30 (SD = 1.87) in the control group, from 8.11 (SD = 2.09) to 10.20 (SD = 2.50) in the booklet group and from 8.36 (SD = 1.88) to 11.81(SD = 2.13 in the booklet + interactive pictorial education group.	After the intervention, the mean knowledge score (range 0–15) increased by 0.27 points in the control group, by 2.9 points in the booklet group and by 3.45 points in the booklet + education group. The mean self‐efficacy score (range: 13–39) increased by 1.33 points in the control group, by 1.98 points in the booklet group and by 4.31 points in the booklet + education group.
						To increase self‐confidence in safe medicine management.		
Parker, R. et al., 2012^61^	Pilot study of usability and acceptability	8 weeks	Adherence was self‐reported. Participants' perceived changes in self‐care ability and quality of life were assessed with an ad hoc designed questionnaire.	A medication reminder device, supported by a remote monitoring service, was installed in participants’ homes. A GP programmed the reminder device according to the daily schedule, in agreement with the patient. If patients ignored the reminder, they received a call reminder by the research team.	Adherence. Participants received both automatic reminders and call reminders to take the right medicine at the right time	To increase adherence.	The self‐reported adherence score increased significantly from 52% (pre) to 81% (post), (*p* = .012). The percentage of participants who considered their self‐care ability excellent increased from 42% to 68% (*p* = .001). Changes in Quality of Life measurements were not significant. Participants found the service acceptable, easy to use and helpful.	In measurements taken after the end of the intervention, the adherence mean score increased by 29% and the perceived self‐care ability mean score increased by 26%. The service was found to be acceptable.
						To increase perceived self‐care ability.		
						To improve quality of life.		
Poureslami, I. et al., 2016^62^	4‐arm RCT.	One single exposure to educational material. Participants were assessed at 3 months	Inhaler technique was assessed with observation, and self‐efficacy in chronic obstructive pulmonary disease (COPD) management was assessed with a validated tool. Knowledge of pulmonary rehabilitation procedures was assessed with an ad hoc tool.	Educational videos on COPD knowledge and self‐management and inhaler tutorial were produced both in Mandarin and in Cantonese with participatory methods. Group A watched a clinician‐led video, group B watched a patient‐led video, group C watched both videos and group D read a leaflet on the same topics.	Knowledge of condition and how to self‐manage knowledge of pulmonary rehabilitation programme (understanding condition, managing symptoms, responding to problems, achieving goals) Knowledge of medicine administration How to use different inhalers Confidence in COPD self‐management.	Increase knowledge of pulmonary rehabilitation procedures	3 months after the intervention, only groups A (clinician video) and B (patient video) had significantly increased mean knowledge of pulmonary rehabilitation procedures, compared with the control group (group A: MD = 2.14; 95% CI = 0.73–3.16; *p* < .05 and group B: MD = 2.22; 95% CI = 0.86–3.30; *p* < .05). The increase in the inhaler technique score (0‐10) was significantly higher in group A (clinician video) (MD = 2.34; 95% CI = 1.34–3.34; *p* < .001), followed by group B (lay video) (MD = 1.92; 95% CI = 0.91–2.93; *p* < .001). Improvement in self‐efficacy was mixed for different items, with all intervention groups showing significantly higher increase in preparedness to manage a COPD exacerbation (*p* < .01) compared with the control group.	After 3 months, patients who watched the clinician‐led video showed the highest increase in knowledge of pulmonary rehabilitation procedures, compared with the control group that read the leaflet. All intervention groups showed improvement in correct use of inhaler compared with the control group). Also, group A (clinician video) showed the greatest improvement. Results for self‐efficacy were mixed for different items.
						Improve inhaler technique.		
						Increase self‐efficacy in COPD management.		
Schulz, R. M. et al., 2011^63^	Prospective cohort study.	12 months	Number of care homes and/or nursing home admissions	Patients received a medication management service coordinated by a health educator with medicines dispensed in a tailored calendar card	Medicine calendar cards to enhance adherence and tailored support in Coordination of care between patients/caregivers, pharmacists and physicians. Addressing problems with adherence.	Reduce care home admissions.	6 out of 273 (2.2%) participants in the intervention group and 40 out of 800 (5%) participants in the control group were admitted to a nursing home at least once during the study period (12 months). Group membership (intervention or control: OR 0.340; 95% CI: 0.119–0.968) was found to be predictive of nursing home admission. Patients who received medicine management services were 66% less likely to be admitted to a nursing home compared with the control group.	Patients who received a medicine management service were 66% less likely to be admitted to a nursing home compared with patients in the control group.
Sidel, V. et al., 1990^64^	2‐arm RCT	6–11 months Assessment at baseline and after the intervention	Medicine‐Related Risk Profiling tool and a Core Questionnaire (ad hoc)	Participants in the intervention group received 2 home visits and phone call follow‐ups by a pharmacist. Participants in the control group received care as usual.	Knowledge of medicines: personalized information on condition and medicines watching out for side effects safe and tidy storage Adherence use of memory aids Communication with healthcare informing HCP before stopping meds The pharmacist reported problems to the GP if necessary (e.g., side effects) and encouraged patients to communicate with HCP	Improve understanding of medicine‐related risk.	After the intervention, no significant difference was found in the overall risk assessment score in the intervention group, compared with the control group, including understanding of medicine‐related risks and attitudes or behaviours related to medicine risk. In the intervention group, the frequency of visits to the outpatient department clinic decreased significantly during the time of the intervention, while it increased in the control group.	No significant difference was found between the control group and the intervention group when the Risk of Medicine‐related profile was re‐assessed after the intervention. A significant difference between the groups was found in change to the frequency of visits to outpatient clinics.
						Encourage change in attitudes towards medicine‐related risk.		
						Reduce medicine‐related problems		
						Increase knowledge of medicines.		
						Reduce cost of care (less frequent outpatient visits)		
Suzuki, R. et al., 2018^65^	Evaluation of the medicine support system.	The mean experimental period for the participants was 91.5 days (SD = 53.9 days).	Adherence was assessed by counting the number of medicine dosages collected from the automatic dispenser	A one‐dosage package medicine support system was installed at participants’ homes and programmed. At the right time, an alarm went off and the medicines came out of the dispenser, to be taken by the patient. If the medicines were not collected, one of the participant's supporters was alerted and invited to call and remind the patient.	Adherence: reminders to take medicines. Preprogrammed medicine dispenser with alarms to support adherence. Patients received additional reminders from family/friends if they failed to take their medicines at the right time.	Increase adherence.	8 out of 10 patient participants collected 100% of doses during the experimental period. The 2 patients who missed doses during the experimental period were able to take their medicines after having been reminded by their supporters. Four patients changed from having their medicines administered by family to self‐administration after using the system.	During the time of the experiment, 100% adherence was reached by 8 out 10 patients.
Wang, C. J. et al., 2013^66^	Pilot study with 2 groups randomly assigned.	2 months. Participants were assessed at baseline and after 10 weeks.	Knowledge, attitudes and behaviours of medication safety were assessed with an ad hoc tool.	Coaching programme based on the Medication Safety Pictorial Guide. Patients in the intervention group received 3 home visits + follow‐up phone calls by trained lay leaders. Patients in the control group received care as usual.	Coaching programme included Knowledge of medicines review of each medicine (shape, colour, dosage, schedule) with patients safe medicine disposal Adherence encouragement to adhere to schedule Strategies to prevent errors checking medicines (when you get them, when you take them and after taking them) Communication with healthcare professionals encouragement to consult the pharmacist for queries. Techniques included Teach back, show back and pictorial information.	Increase knowledge of medication safety.	At 10 weeks, patients in the intervention group had a significantly increased mean score in knowledge of medication safety compared with baseline (from to 5.6, (SD = 1.6) to 7.0, (SD = 0.8) *p* < .001). A significantly higher percentage of patients in the intervention group recorded positive changes across 3 medication safety behaviours: a) checking the medications received (7 patients in the intervention group and 1 patient in the control group started checking the medication received), b) checking before taking medicines (9 patients in the intervention group, 2 patients in the control group) and c) disposing of surplus drugs correctly (14 patients in the intervention group, 6 patients in the control group). Within‐group and between‐group differences in the attitude scores were not significant	At 10 weeks, the mean score in knowledge of medication safety for the intervention group was significantly higher compared with the control group (*p* < .05). A significantly higher percentage of patients in the intervention group showed positive changes in behaviour in relation to medicine check and disposal.
						Improve attitude towards medication safety.		
						Encourage changes in medication safety behaviours.		
Whittaker, C. F. et al., 2017^67^	Quasi experimental design with 2 non randomized groups.	The time required for completing the game/reading leaflets was not specified. Participants were assessed before the intervention, immediately after the intervention and at 30 days.	Knowledge of medicines' poisoning risk and intended behaviour in relation to medications' poisoning risk was assessed using a Medicine IQ index.	Interactive group game on medicines poison prevention delivered by a pharmacist at older adults’ centres. Participants were split into two groups. The first group played an interactive jeopardy‐style game designed by the Poison Centre. The second group read leaflets on medication safety and medicines’ poison prevention.	Content addressed in the game and the leaflets included Knowledge of medicines Importance of checking medicines labels before use Importance of keeping a full list of medicines Response to emergency Emergency contact for advice on medicine safety	Increase knowledge of medication safety and poison prevention resources,	In the assessment after the intervention, a higher increase in the mean Medicine IQ score was found in participants who played the game compared with participants who read a brochure. Among game players, the median Medicine IQ increased from 9 (interquartile range, IQR: 6, 9) to 11 (IQR: 9, 12) among participants who read the leaflet; the Medicine IQ increased from 7.5 (IQR: 6, 8) to 8 (IQR: 5, 10).	After the intervention, patients in the game group obtained higher Medicine IQ scores compared with the ones who read the leaflet.
						Induce changes in planned behaviours in response to poisoning risk		
Wong, A. K. C. et al., 2019^68^	2‐arm RCT	3‐month education programme. Measures were taken before and after the intervention.	Self‐efficacy: General Self‐efficacy Scale. Quality of Life and Activities of Daily life: 12‐item Short Form Health Survey version, ADL Modified Barthel Index, IADL Lawton scale. Adherence: Adherence to Refills and Medications Scale. Number of visits/admissions to healthcare.	Comprehensive assessment (Omaha system) and education programme delivered by nurses at home and with follow‐up calls. Patients in the control group received placebo calls and usual care.	Programme included self‐care goal‐setting and tailored education on adherence At follow‐up visits and calls, a nurse and a community worker evaluated progress in self‐care and adherence and made referrals when necessary.	Increase adherence.	In the intervention group, the scores of self‐efficacy (*p* = .049), ADL (*p* = .012), IADL (*p* = .021) and physical components of QoL (*p* < .001) increased significantly between the baseline and the postintervention assessment. Compared with the control group, adherence improved significantly in the intervention group after the intervention (*p* < .001), while access to healthcare decreased significantly (*p* = .016).	After the intervention, patients in the intervention group reported a significant increase in self‐efficacy and quality‐of‐life measures. Compared with the control group, intervention patients’ adherence increased significantly and their use of healthcare service decreased significantly.
						Increase self‐efficacy.		
						Improve quality of life/instrumental activities of daily living.		
						Reduce access to healthcare services (GP, outpatient and hospital visits).		
Poonprapai, P. et al., 2022^69^	2‐arm RCT	3 months. Adherence measured at baseline and at 3, 6 and 9 months.	Value of glycosylated haemoglobin and blood pressure Family behaviour in diabetes care: ad hoc tool knowledge of diabetes: ad hoc tool Diabetes self‐care skills: ad hoc tool. Adherence (pill count) (patient)	Educational intervention delivered via App. Family members in the intervention group received daily educational infographics and quiz on diabetes self‐care and medicine management to share with patients. Family member could access a pharmacist via App at any time. The control group received care as usual.	Content shared via app included Knowledge of conditions and how to self‐manage information on diabetes; self‐management good practices (e.g., nutrition, physical activity, foot care); management of comorbidities Knowledge of medicines: prevention of adverse reactions Adherence medicine adherence strategies reminders for medicine taking and appointments Motivation • Motivating messages to improve support	Improve adherence.	Diabetes clinical outcomes improved in the intervention group, with values of glycosylated haemoglobin and blood pressure significantly declining from baseline to 9 months (*p* < .001). And, changes in values were significantly different between the intervention group and the control group (*p* ≤ .001). At 9 months, diabetes control‐related measures (family behaviour, patients’ knowledge, caregivers’ knowledge and patient’ self‐management) improved significantly in the intervention group (*p* < .05), with changes in scores differing significantly between the intervention and control groups in all items (*p* ≤ .001). At 9 months, adherence (scale range 0‐100) increased from 87.17 (SD = 2.04) to 91.41 SD = 3.57) in the intervention group and from 87.28 SD = 2.29 to 88.47 (SD = 2.54) in the control group. The increase in adherence at 3 and 9 months was higher in the intervention group, and the difference in increase between the intervention group and the control group was significant (*p* < .001)	At 9 months, the mean adherence score increased by 4.24% in the intervention group and by 1.19% in the control group. The mean change in patients' knowledge was MD = 2.86 (SD = 1.99, *p* < .001) in the intervention group and MD = 0.63 (SD = 2.23, *p* < .014) in the control group. The mean change in carers’ knowledge was MD = 3.15 (SD = 2.86, *p* < .001) in the intervention group and MD = 0.29 (SD = 1.62, *p* < .114) in the control group. Both diabetes control measures and diabetes clinical outcomes improved in the intervention group.
						Improve clinical outcome of diabetes		
						Improve family support in diabetes management.		
						Improve diabetes knowledge and diabetes self‐care abilities.		
Zhang, S. et al., 2022^70^	Prospective cohort study	All enrolled patients were followed up for 3 months.	Adherence: Morisky Green Levine Medication Adherence Scale (MGLS range 0–4, the lower the better) Health‐related Quality of Life EuroQol 5 Dimension scale (EQ‐5D) and EuroQol‐visual analog scale EQ‐VAS	Participants received medicines review and education by a pharmacist at home. After assessing adherence, medicine‐related problems, health‐related quality of life and knowledge of medicines and conditions, the pharmacist proposed solutions (to the patient or GP), delivered tailored education and planned follow‐up appointments.	Tailored education included Knowledge of conditions tailored information to enhance understanding of medications and conditions Adherence discussion of strategies to enhance adherence	Improve adherence.	The average number of drug‐related problems per patient decreased significantly (*p* < .001) from 0.88 (SD = 1.29) at baseline to 0.4 (SD = 0.94) at the 3‐month follow‐up. Adherence significantly increased, with the mean MGLS score reduced from 1.42 (SD = 1.35) to 0.85 (SD = 1.14), (*p* < .001). Health‐Related Quality Measured by EQ‐5D increased significantly from a mean score of 0.75 (SD = 0.10) to 0.78 (SD = 0.08) at follow‐up (*p* < .001), as well as the Q‐VAS measure, which improved from a mean of 70 (SD = 13.61) to 77.65 (SD = 11.18) *p* < .001.	The average number of medicine‐related problems per patient decreased from 0.88 to 0.4. The mean adherence score increased by 0.57 points in the MLGS scale (0–4). Health‐related quality‐of‐life measures increased by 0.03 points in the EQ‐5D scale (range not reported) and by 7.65 points in the ED‐VAS scale (range: 0–100)
						Assess medicine‐related problems.		
						Encourage medicines' optimization.		
						Improve quality of life.		
Falamić, S. et al., 2021^71^	2‐arm RCT	45 min + 20 min monthly follow‐up for 6 months. Health‐Related Quality of Life and satisfaction with Warfarin therapy was assessed at 6 months only.	Health‐Related Quality of Life and satisfaction with Warfarin therapy (Cro‐DASS questionnaire measuring limitations, hassles and burden and psychological impact)	Participants in the intervention group received medicines review, warfarin optimization (e.g., resolving problems with medicines' interactions and nonadherence issues), education on warfarin therapy and follow‐up plan. Participants in the control group received care as usual.	Knowledge of medicines tailored education on warfarin therapy Adherence compliance aid (pill box).	Increase quality of life of patients taking anti‐coagulants.	At the 6‐month assessment, the median score of Health‐Related Quality of Life measured with the Cro‐DASS scale (range: 25–175, with lower scores indicating a better quality of life) was 66.0 (63.0–69.0) *p* < .001 in the intervention group and 86.5 (77.0–94.0) in the control group. Differences between the two randomized groups were significant (*p* < .001) in all domains (limitations, hassles and burden and psychological impacts) of Health‐Related Quality of Life.	At 6 months, the mean Health‐Related Quality of Life and satisfaction with Warfarin therapy score in the intervention group was 20.5 points lower (range: 25–175, the lower the better) than that of the control group, indicating higher satisfaction with Warfarin.
						Address problems associated with anti‐coagulant use.		
						Encourage medicines' optimization.		

Abbreviations: HCP, healthcare professional; RCT, randomized‐controlled trial.

### 3.6 Intervention aims and effect

In the majority of the interventions (n = 19), improving patients’ adherence was one of the aims (*n* = 13) or the only aim (*n* = 6).^43^, ^46^, ^48^, ^49^, ^57^, ^65^ Of all interventions aiming to improve adherence, four did not report complete outcome measures^41^, ^42^, ^50^, ^65^ and one^45^ reported that changes in adherence were not significant. All the remaining studies (*n* = 14) reported significant differences in adherence scores, either compared with baseline, or between the intervention and control group(s) in measurements taken after the intervention (see Table 2). One of those^49^ found that although in the intervention group the adherence score increased significantly at 4 weeks, the increase was not sustained at the 5‐month follow‐up.

Among the papers where interventions sought to improve knowledge (*n* = 17), three articles did not report outcome measures,^41^, ^42^, ^50^ ten reported a significant increase in participants’ measures of knowledge taken after the intervention compared with baseline or between control and intervention groups,^42^, ^45^, ^54^, ^58^, ^59^, ^60^, ^62^, ^66^, ^67^, ^69^ while four articles^39^, ^40^, ^51^, ^64^ reported that changes in knowledge were not significant (Table 2).

All the interventions aimed at increasing self‐efficacy^53^, ^54^, ^55^, ^58^, ^59^, ^60^, ^61^, ^62^, ^68^ (*n* = 9) reported significant improvements in the measurements taken after the intervention, for all or some of the items assessed (Table 2).

Of the four studies that aimed to increase communication with HCPs,^42^, ^47^, ^54^, ^56^ one reported that after the intervention, participants started conversations with physicians,^47^ one registered increased interactions with pharmacists,^54^ one found that more patients planned to bring an updated list of medicines at their next visit to a pharmacist^56^ and one did not report outcome measures.^42^

All interventions assessed in this review aimed to achieve a change either in participants’ knowledge, attitudes, behaviours, self‐efficacy and quality of life or a combination of them, but some also reported systemic goals,^39^, ^52^, ^63^, ^68^ such as reducing admissions to care homes or hospital. Significant changes reported by those included decrease in cost of care after a home medicines review^40^ and decrease in healthcare use after an education programme.^68^ Finally, a prospective cohort study^63^ found that participants using medication calendar cards were 66% less likely to be admitted to a nursing home than nonusers.

### 3.7 Intervention characteristics

The six interventions that solely aimed to increase adherence were either based on new dosage packaging,^57^ dispensing systems with reminders,^46^, ^63^, ^65^ new ways to provide administration instructions^43^ or coaching programmes to enable patients to develop medicine‐taking routines.^48^, ^49^

Most interventions that sought to improve knowledge^39^, ^41^, ^42^, ^45^, ^50^, ^51^, ^60^, ^64^, ^66^ (*n* = 9) aimed to increase the general understanding of medicines and medicine regimens, for example, reasons for taking them, dosage, timing and additional instructions. Some also aimed to enhance medicines' safety knowledge.^42^, ^60^, ^66^ Patients, for example, learned how to recognize side effects,^60^ avoid mistakes in dosing and timing and how to repeatedly check their medicines,^66^ including the medicines received from the pharmacy.

A number of interventions focused on increasing participants’ knowledge of specific topics (*n* = 7) such as self‐management and therapy of specific conditions,^59^, ^62^, ^69^ risk associated with medicine poisoning,^67^ risk of medicines and alcohol interactions and^58^ anticholinergic medicine‐associated risks.^47^ Finally, one intervention aimed at improving knowledge of the role of pharmacists in medicine management.^54^

Two studies compared the effectiveness of different educational materials, such as playing a game^67^ or watching a video tutorial^62^ versus reading leaflets on the same topic, with the more interactive mode showing better results.

### 3.8 Mapping resilience abilities

To determine which resilience abilities^36^ the interventions potentially addressed, we explored whether they supported patients to (a) *learn*, for example, about their medicines and conditions or by previous experiences of their care, (b) *monitor*, for example, their health and the impact of medications, (c) *anticipate*, for example, problems in accessing or managing medicines and (d) *respond* to unexpected events. The results are presented in Table 3.

**Table 3:** Resilience abilities supported by the intervention.

Authors, year	Intervention type	Resilience abilities
Bernsten, C. et al., 2001^39^	Medicines review and one‐to‐one education	*Learning* about medicines and how to store them. *Learning* how to use compliance tools
Sturgess, I. K. et al., 2003^40^	Medicines review and one‐to‐one education	*Learning* about medicines and how to store them. Learning how to use compliance tools
Akers, J. L. et al., 2019^41^	Medicines review and one‐to‐one coaching	*Learning* about medicines. *Monitoring* problems with medicines through a shared list and follow‐up
Benoit, M. L., 2016^42^	Group educational programme with optional medicines review	*Learning* about medicines and how to store and dispose them. *Learning* about medication safety. *Learning* how to keep a medication list and use tools to support adherence. *Learning* to communicate with HCP.
Bilotta, C. et al., 2011^43^	Administration instructions	*Learning* about when to take medicines.
Fulmer, T. et al., 1999^44^	Medicine reminder calls	Unable to map. The intervention prompts medicine administration.
Griffiths, R. et al., 2004^45^	Medicines review and one‐to‐one education	*Learning* what medicines are for and how to take them.
Hayes, T. L. et al., 2009^46^	Medication reminding system	Unable to map. The intervention prompts medicine administration.
Holden, R. J. et al., 2020^47^	APP with assessment tool and education on anticholinergics	*Learning* about anticholinergic medicine risks. *Responding* to risks by starting a conversation with HCP about potential de‐prescribing.
Insel, K. C., Cole, K. L., 2005^48^	One‐to‐one coaching and adherence monitoring tool	*Monitoring* own adherence using traceable context/cues
Insel K. C., et al., 2016^49^	One‐to‐one education and coaching sessions	*Learning* about condition and antihypertensive medicines. Provide *monitoring* tools to maximize adherence
Lagerin, A. et al., 2014^50^	Assessment and one‐to‐one education sessions	*Learning* about medicines.
LeBlanc, R. G., Choi, J., 2015^51^	Assessment and one‐to‐one education sessions	*Learning* about their medicines (aim, dose, timing) and generating an updated medication list
Lenaghan, E. et al., 2007^52^	Medicines review and one‐to‐one education	*Learning* about medicines.
Martin, D. et al., 2012^53^	Pictorial administration instructions	*Learning* about medicines and doses
Martin, B. A. et al., 2016^54^	Small group interactive educational programme	*Learning* about medicines, learning about pharmacist role. *Responding* to medicine‐related concerns by starting a conversation with HCP.
Meyer, M. et al., 2021^55^	Medicines review and one‐to‐one education	*Monitoring* health such as tracking blood sugar. Providing an action plan and tracking log.
		*Learning* how to use medicines, the effects of medicines and practical skills such as filling pill boxes and disposing of medicines. *Learning* about the importance of keeping a list.
Miller, M. J. et al., 2008^56^	Group educational presentation	*Learning* about medicines. *Learning* how to communicate about medicines with the pharmacist. *Learning* to keep and carry an updated medication list.
Murray, M. et al., 1993^57^	Dose packaging service	Unable to map. Intervention prompts medicine administration.
Neafsey, P. J. et al., 2001^58^	Educational programme via interactive software	*Learning* about medicine interactions and interactions with alcohol.
Park, E., Kim, J., 2016^59^	One‐to‐one education and coaching sessions	*Learning* about hypertension and hypertensive medicines
Park, M., 2011^60^	Small group pictorial educational programme	*Learning* about medication safety (how to dose medicines, recognize side effects and avoid drug interactions). *Learning* how to read medicine leaflets.
Parker, R. et al., 2012^61^	Medication reminding system	Unable to map. Intervention prompts medicine administration
Poureslami, I. et al., 2016^62^	Individual educational programme (video)	*Learning* about condition and *learning* how to use inhaler
Schulz, R. M. et al., 2011^63^	Dose packaging with assistance	Unable to map. Intervention prompts medicines taking by changing the way they are dispensed.
Sidel, V. et al., 1990^64^	One‐to‐one education sessions	*Learning* about medicines through personalized information plus encouragement to manage them well. *Learning* about the importance of communicating with clinicians.
Suzuki, R. et al., 2018^65^	Medication reminder system	Unable to map. Intervention prompts medicine taking.
Wang, C. J. et al., 2013^66^	One‐to‐one education and coaching sessions	*Learning* about medication safety, medication schedules, how to store and dispose medicines, learning how to communicate with pharmacist.
		*Monitoring* supply, checking when you get them, before taking them, when you take them
Whittaker, C. F. et al., 2017^67^	Group educational programme	*Learning* about medicines' poisoning risk. *Learning* about the importance of having a medication list.
Wong, A. K. C. et al., 2019^68^	Assessment, one‐to‐one education and coaching sessions	*Learning* about self‐care and adherence.
Poonprapai, P. et al., 2022^69^	Infographics shared via App to family carers	Learning about diabetes and diabetes self‐care and therapy
Zhang, S. et al., 2022^70^	Medicines review and education	*Learning* about conditions and medicines
Falamić, S. et al., 2021^71^	One‐to‐one medicines review and education	*Learning* about anti‐coagulant therapy.

Abbreviation: HCP, healthcare professional.

**Figure 2:**
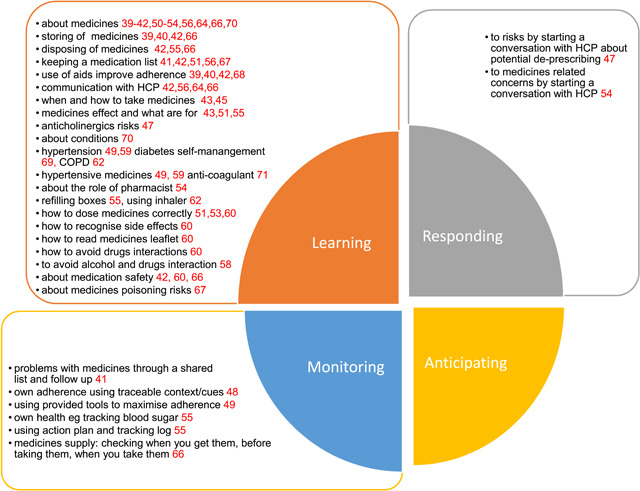
Resilience abilities addressed by interventions and topics covered. HCP, healthcare professional.

Most studies (*n* = 26) supported participants in *learning* about medicines and conditions. Some focused on preventing risks associated with medicine use. This included *learning* how to use adherence aids, to avoid errors, how to keep updated lists of medicines, how to store and dispose of medicines and how to communicate effectively with HCPs about medicines. Other studies (*n* = 5) supported patients’ ability to *monitor* medicine supply,^66^ problems with medicines through a list shared with HCPs,^41^ adherence through self‐monitoring tools^48^, ^49^ and tracking logs.^55^ Two studies supported patients' abilities to *respond* to medicine concerns or risks through starting a conversation with a GP,^54^ including about potential de‐prescribing.^47^ Details of the topics covered and resilience abilities addressed are shown in Figure 2.

## 4 DISCUSSION

This rapid review identified 33 articles describing interventions to support community‐dwelling older people to self‐manage their medicines. Overall, our findings indicate that the majority focussed on enhancing adherence and knowledge of medicines. The studies included in this review that reported outcomes in more detail were not studies that focused on enhancing the medication safety aspects of self‐management. Whilst there are a few examples of what types of intervention could be delivered, the outcomes of these are unclear.

We have identified clear gaps, based on the evidence about patient experiences with their medicines and on the proactive strategies that they implement.^28^ These are as follows:

− Interventions that support people to navigate the complexity of the medicine management system (e.g., where there are multiple prescribers and frequent changes).
− Interventions that prepare people to anticipate and respond to errors in the system (e.g., receiving incorrect medicines).
− Interventions that support people to detect problems in their medicine self‐management abilities, recognize when they may be deteriorating and ask for help.

Our review found that medicine self‐management interventions do not generally attempt to augment patients’ proactive safety roles. Interventions aimed at other areas of healthcare have attempted to enhance patient roles. Lawton et al.,^72^ for example, evaluated an intervention to support patients to report safety concerns in hospital. They concluded that, while patients were willing and able to report concerns, staff also needed support to respond to patient feedback. More recent work has indicated that people play important roles in the care that they receive for chronic healthcare conditions. Systematic review has demonstrated that a lack of involvement in care is not a desired state for patients; rather, it is one that is forced on them by the system and the context in which care is provided.^73^ Patient activation as a concept has gained traction since Hibbard et al's measure was first introduced in 2004.^74^ Our review, however, shows that little has been explored to support patient activation to be proactive partners in the safe use of medicines.

Previous work has highlighted how the safety‐critical role of patients and carers is overlooked^75^ and interventions supporting patients to monitor the system and respond to safety risks would augment their role. However, careful consideration must be given to ensure that such interventions do not entrench existing inequities in the system. Those who struggle to manage medicines because, for example, they are not first‐language English speakers,^76^ or because they do not have the confidence to ask questions, must not be further disadvantaged by an intervention that does not take their needs into account.^77^

### 4.1 Patients’ resilience abilities

Fylan and colleagues^28^ outlined how patients and their informal carers are under‐recognized sources of system resilience. Interventions that enhance patients’ abilities to learn, respond, anticipate and monitor have the potential to enhance safety overall in the medicine management system. Interventions in this review mainly supported patients in learning about their medicines. Only two interventions were found that supported the ability to respond, starting conversations with a HCP to discuss concerns about medicines. Interventions explicitly addressing the ability to anticipate were not found in this review. In a few interventions, though, it was assumed that untidy medicine storage would increase errors, so patients were trained to store and dispose of medicines correctly, to prevent confusion. Only a minority of interventions explicitly addressed the ability to monitor and in only one intervention patients were coached to carefully monitor their supply. This included checking that the medicines received from the pharmacy were correct. Being aware that errors can happen in the system, patients may become more vigilant and this could contribute towards preventing adverse events.

### 4.2 Proactive roles of patients

O′Hara and colleagues^78^ discussed how patients bolster safety in complex and unpredictable systems through their actions and adjustments. Being the only actors in the system who see the whole pathway, patients are in fact in a unique position to recognize safety risks across sectors and organizations. Medicines are an aspect of care that patients use independently, after the initial act of prescribing and subsequent reviews. Medicines cost the NHS in England £17billion per year and there are startlingly high levels of medication error. It is interesting then that few interventions target how people ‘manage’ their medicines rather than how they ‘take’ their medicines. Using the Resilient Healthcare theory^27^ to develop an intervention around medicine self‐management would support patients in being proactive in managing their regimens and their interactions with healthcare. Managing multiple medicines requires patients to be adaptable to their changing medicine regimens and to be vigilant in a system that might pose risks to their safety. Therefore, an intervention to support them must support that adaptability, alert them to the need to monitor how the system functions and should change as their needs, and regimens change. Such an intervention needs to enable the patient to focus on what is important to them. It also needs to support them to embed medicine taking in the context of their daily lives, for example, helping them to schedule medicines into their daily routines and breaking the ‘jobs’ required by medicine self‐management into manageable tasks. Interventions that enable this would align with the NHS view of ‘supported self‐management’, which recognizes people's skills and strength in addition to their needs.

### 4.3 Informal carers

Only in 7 out 33 articles targeted informal carers as well as patients; only one was delivered to the family network of supporters, instead of to patients.^69^ Such a low percentage of interventions could be explained by the fact that articles specifically targeting patients living with dementia or cognitive diseases were excluded. The number or ratio of informal carers involved in the intervention was not reported by three out of seven and only two studies reported more information on the different roles played by patients and their informal carers.^65^, ^69^ This suggests that patients and their informal carers might have been treated as interchangeable in the remaining five studies. The relationship between older patients and their support network has not been explored in any of the articles found in this review. Research conducted with patients with long‐term conditions, however, indicates that such relations are complex and ever changing, with the distribution of tasks constantly re‐negotiated. Interventions are needed that take into account the multiple and ever‐changing roles that patient support networks play in medicine management.

### 4.4 Frailty, multimorbidity and polypharmacy

Only one intervention was found that specifically targeted older people with frailty. People living with frailty have been found to be particularly vulnerable to the consequences of inappropriate polypharmacy and medicine errors, and interventions can support patient roles in how medicines are managed in this population.^79^ More interventions are required to support this population to manage polypharmacy, recognize when their symptoms might be deteriorating and avoid unnecessary stressors that can cause further deterioration.^79^, ^80^

Complex medicine regimens, polypharmacy and multimorbidity in older populations have been associated with negative health outcomes.^13^, ^16^ Conversely, it was surprising to find that only 7 interventions targeted people either taking three or more medicines or living with multiple long‐term conditions. We recommend that in the future, interventions to support self‐management of medicines prioritize frail and/or multimorbid populations dealing with complex medicine regimens.

### 4.5 Defining medicine self‐management

A new, more comprehensive understanding of medicine self‐management components, co‐designed with patients and their network of supporters, is needed to design interventions that are grounded in the knowledge of the processes involved and embody the patient perspective.^29^ Informed by the literature in this review and by our patient advisory group, we propose the following (Table 4) as components for safe self‐management of medicines:

**Table 4:** Self‐management of medicines—Key components.

Knowledge of medicines	To understand what medicines have been prescribed and why, including how and when to take them
Managing supply	To understand how to access supply (prescription journey, repeat prescriptions, impact of changes in medicines, knowing when is the right time to order, dealing with inputs from different prescribers—e.g., GP and specialists)
	To monitor medicine supply, anticipating problems and knowing how to respond to unexpected events (delays, errors, changes not actioned in prescription).
Monitoring how you feel	To monitor medicines' effects and side effects, especially when changes are introduced and knowing how to respond (e.g., knowing what to do in case of an adverse event).
Ensuring that medicines are taken as instructed	To develop ways to monitor that medicines are taken as instructed (self‐monitoring of adherence).
	To develop ways to overcome barriers to adherence (e.g., special containers, calendar reminders, daily routines), anticipating where adhering might be difficult, for example, when routines are disrupted.
Communication with healthcare professionals	To communicate effectively with healthcare professionals about medicines, including identifying and targeting the relevant professional, feeling confident about asking questions and feeling empowered to make informed decisions.
Self‐monitoring and involving other people	To monitor their own self‐management skills, learning to know where help will be needed and delegating tasks to others, if self‐managing becomes difficult.
	To be able to share instructions and information related to their medicines with people (family, friends, professional carers) willing to help.
Adaptability	To be able to cope with ever‐changing circumstances (change in symptoms, in doses, brand, timing) and constantly adjust.

Managing many medicines can be challenging and requires a wide range of skills. Each time a new condition is diagnosed, or a symptom deteriorates, patients need to adapt the way they cope. Living with multiple long‐term conditions demands increasing efforts to organize and remember multiple medications, to manage healthcare appointments, to perform self‐care and change lifestyles and routines.^81^, ^82^, ^83^ Interventions that can be adapted over time are therefore preferable, as they will be able to provide support both when people's needs change (e.g., loss of dexterity or vision) and when new challenges or opportunities arise in the healthcare system (e.g., medical practices accepting only medicine orders online). An adaptive design could be explored to enhance responsiveness.^84^

In the interventions identified by this review, education on medicines and condition self‐management was delivered in a variety of ways, including using software, video tutorials and Apps. Future interventions need to carefully consider the impact of digital exclusion on the most vulnerable in the older population (e.g., over 70, living alone, on lower income),^85^ to avoid making health inequalities worse. The use of participatory methods would, in our view, ensure that the content addressed by the intervention is relevant^86^ and the mode of delivery is appropriate to people's abilities and circumstances.

Finally, an intervention to support older patients around their medicines, in the first place, needs to avoid placing additional burden on their shoulders, starting from considering what patients and their networks already do and respecting them for ‘what they do’.^87^ Medicine regimens should be person‐centred to take into account patient values and preferences as well as the effects of multiple comorbidities and social circumstances.^88^

### 4.6 Strengths and limitations

To our knowledge, this is the first rapid review to characterize interventions aimed at enhancing how older patients self‐manage their medicines from a Resilient Healthcare perspective. A recent systematic review looked at the resilient abilities addressed by interventions to support medicine self‐management at home, but only included studies targeting patients living with dementia or cognitive impairment.^29^ A previous review investigated RCT interventions to support older people's ability to take medicines and adherence, when more than 4 medicines were prescribed.^89^ This is the first review to include both a variety of study designs and interventions addressing a range of aspects related to medicine self‐management.

This review did not include a citations search or grey literature. A quality assessment and risk of bias assessment were not performed because they were not necessary to meet the study aim. A narrative approach was chosen to describe the results and no quality assessment was conducted on the interventions included. Nevertheless, this review offers useful insight into the aspects of medicine self‐management targeted by interventions aimed at an older population. Adopting a Resilient Healthcare perspective, finally, this review offers a new definition of medicine self‐management that we hope could inform interventions that enhance older people's resilience capabilities.

## 5 CONCLUSION

Few interventions were found that address the full range of challenges that older people face in self‐managing medicines and only one that specifically targets older people living with frailty. Most identify deficiencies within the patient, rather than preparing them for problems inherent in the medicine management system, and address their knowledge, attitudes or behaviour around medicines to effect a change. Little is known about how patients, informal carers and HCPs could better cooperate to improve the self‐management of medicines at home. Patient safety may be enhanced by developing interventions that, on the one hand, consider the complexity and the variety of tasks and skills involved in medicine self‐management and, on the other, recognize the role that patients and their support network play in maintaining safety.

## AUTHOR CONTRIBUTIONS

Giorgia Previdoli, V‐Lin Cheong, David Alldred, Justine Tomlinson, Savi Tyndale‐Biscoe, Jonathan Silcock, Daniel Okeowo and Beth Fylan made substantial contributions to the drafting of the manuscript, defining the research question and identifying the key words. Giorgia Previdoli, V‐Lin Cheong, David Alldred Jonathan Silcock, Daniel Okeowo and Beth Fylan took part in the screening process. Giorgia Previdoli, Beth Fylan and Justine Tomlinson mapped the resilience abilities. All authors reviewed and approved the final version of the manuscript.

## CONFLICT OF INTEREST STATEMENT

The authors declare no conflicts of interest.

## Supporting information

Supporting information.

Supporting information.

## Data Availability

Data sharing is not applicable to this article as no new data were created or analysed in this study.

## References

[1] Population Division, Department of Economic and Social Affairs, United Nations. *World Population Ageing 2019* (ST/ESA/SER.A/444); 2020. Accessed February 2, 2023. https://www.un.org/en/development/desa/population/publications/pdf/ageing/WorldPopulationAgeing2019-Report.pdf

[2] Office for National Statistics . Overview of the UK population: January 2021. An overview of the UK population in 2019 (before the coronavirus (COVID‐19) pandemic): how it has changed, why it has changed and how it is projected to change in the future. 2021. Accessed February 2, 2023. https://www.ons.gov.uk/peoplepopulationandcommunity/populationandmigration/populationestimates/articles/overviewoftheukpopulation/january2021

[3] Barnett K , Mercer SW , Norbury M , Watt G , Wyke S , Guthrie B . Epidemiology of multimorbidity and implications for health care, research, and medical education: a cross‐sectional study. Lancet. 2012;380(9836):37‐43. 10.1016/S0140-6736(12)60240-2 22579043

[4] Fabbri E , An Y , Zoli M , et al. Aging and the burden of multimorbidity: associations with inflammatory and anabolic hormonal biomarkers. J Gerontol Ser A. 2015;70(1):63‐70. 10.1093/gerona/glu127 PMC4296167 25104822

[5] Fortin M , Lapointe L , Hudon C , Vanasse A , Ntetu AL , Maltais D . Multimorbidity and quality of life in primary care: a systematic review. Health Qual Life Outcomes. 2004;2(1):1‐12. 10.1186/1477-7525-2-51 15380021 PMC526383

[6] Wang L , Si L , Cocker F , Palmer AJ , Sanderson K . A systematic review of cost‐of‐illness studies of multimorbidity. Appl Health Econ Health Policy. 2018;16(1):15‐29. 10.1007/s40258-017-0346-6 28856585

[7] Masnoon N , Shakib S , Kalisch‐Ellett L , Caughey GE . What is polypharmacy? A systematic review of definitions. BMC Geriatr. 2017;17(1):230. 10.1186/s12877-017-0621-2 29017448 PMC5635569

[8] Midão L , Giardini A , Menditto E , Kardas P , Costa E . Polypharmacy prevalence among older adults based on the survey of health, ageing and retirement in Europe. Arch Gerontol Geriat. 2018;78:213‐220. 10.1016/j.archger.2018.06.018 30015057

[9] Morin L , Johnell K , Laroche M‐L , Fastbom J , Wastesson JW . The epidemiology of polypharmacy in older adults: register‐based prospective cohort study. Clin Epidemiol. 2018;10:289‐298. 10.2147/CLEP.S153458 29559811 PMC5856059

[10] Young EH , Pan S , Yap AG , Reveles KR , Bhakta K . Polypharmacy prevalence in older adults seen in United States physician offices from 2009 to 2016. PLoS One. 2021;16(8):e0255642. 10.1371/journal.pone.0255642 34343225 PMC8330900

[11] Stewart D , Mair A , Wilson M , et al. Guidance to manage inappropriate polypharmacy in older people: systematic review and future developments. Expert Opin Drug Saf. 2017;16(2):203‐213. 10.1080/14740338.2017.1265503 27885844

[12] Medicines Directorate, Department of Health and Social Services . DoHaSSM. *Good for you, good for us, good for everybody. A plan to reduce overprescribing to make patient care better and safer, support the NHS, and reduce carbon emissions*. 2021. Accessed 2 February 2023. https://assets.publishing.service.gov.uk/government/uploads/system/uploads/attachment_data/file/1019475/good-for-you-good-for-us-good-for-everybody.pdf

[13] Wastesson JW , Morin L , Tan ECK , Johnell K . An update on the clinical consequences of polypharmacy in older adults: a narrative review. Expert Opin Drug Saf. 2018;17(12):1185‐1196. 10.1080/14740338.2018.1546841 30540223

[14] Dijkstra NE , Sino CGM , Schuurmans MJ , Schoonhoven L , Heerdink ER . Medication self‐management: considerations and decisions by older people living at home. Res Soc Admin Pharm. 2022;18(3):2410‐2423. 10.1016/j.sapharm.2020.09.004 33627223

[15] George J , Phun YT , Bailey MJ , Kong DC , Stewart K . Development and validation of the medication regimen complexity index. Ann Pharmacother. 2004;38(9):1369‐1376. 10.1345/aph.1D479 15266038

[16] Wimmer BC , Bell JS , Fastbom J , Wiese MD , Johnell K . Medication regimen complexity and polypharmacy as factors associated with all‐cause mortality in older people: a population‐based cohort study. Ann Pharmacother. 2016;50(2):89‐95. 10.1177/1060028015621071 26681444 PMC4714103

[17] Schoonover H , Corbett CF , Weeks DL , Willson MN , Setter SM . Predicting potential postdischarge adverse drug events and 30‐day unplanned hospital readmissions from medication regimen complexity. J Patient Saf. 2014;10(4):186‐191. 10.1097/PTS.0000000000000067 25408236

[18] Clegg A , Young J , Iliffe S , Rikkert MO , Rockwood K . Frailty in elderly people. Lancet. 2013;381(9868):752‐762. 10.1016/S0140-6736(12)62167-9 23395245 PMC4098658

[19] Collard RM , Boter H , Schoevers RA , Oude Voshaar RC . Prevalence of frailty in community‐dwelling older persons: a systematic review. J Am Geriatr Soc. 2012;60(8):1487‐1492. 10.1111/j.1532-5415.2012.04054.x 22881367

[20] Bailey SC , Oramasionwu CU , Wolf MS . Rethinking adherence: a health literacy‐informed model of medication self‐management. J Health Commun. 2013;18:20‐30. 10.1080/10810730.2013.825672 PMC3814610 24093342

[21] Howell EH , Senapati A , Hsich E , Gorodeski EZ . Medication self‐management skills and cognitive impairment in older adults hospitalized for heart failure: a cross‐sectional study. SAGE Open Med. 2017;5:2050312117700301. 10.1177/2050312117700301 28540048 PMC5433792

[22] Maidment I , Lawson S , Wong G , et al. Towards an understanding of the burdens of medication management affecting older people: the MEMORABLE realist synthesis. BMC Geriatr. 2020;20(1):183. 10.1186/s12877-020-01568-x 32498672 PMC7272211

[23] Schafheutle EI , Fegan T , Ashcroft DM . Exploring medicines management by COPD patients and their social networks after hospital discharge. Int J Clin Pharm. 2018;40(5):1019‐1029. 10.1007/s11096-018-0688-7 30056568 PMC6208597

[24] Cheraghi‐Sohi S , Jeffries M , Stevenson F , et al. The influence of personal communities on the self‐management of medication taking: a wider exploration of medicine work. Chronic Illn. 2015;11(2):77‐92. 10.1177/1742395314537841 24920009

[25] Lawson S , Mullan J , Wong G , et al. Family carers’ experiences of managing older relative's medications: insights from the MEMORABLE study. Patient Educ Couns. 2022;105(7):2573‐2580. 10.1016/j.pec.2021.12.017 35016779

[26] Tomlinson J , Silcock J , Smith H , Karban K , Fylan B . Post‐discharge medicines management: the experiences, perceptions and roles of older people and their family carers. Health Expect. 2020;23(6):1603‐1613. 10.1111/hex.13145 33063445 PMC7752204

[27] Braithwaite J , Wears RL , Hollnagel E . Resilient Health Care: turning patient safety on its head. Int J Qual Health Care. 2015;27(5):418‐420. 10.1093/intqhc/mzv063 26294709

[28] Fylan B , Armitage G , Naylor D , Blenkinsopp A . A qualitative study of patient involvement in medicines management after hospital discharge: an under‐recognised source of systems resilience. BMJ Qual Saf. 2018;27(7):539‐546. 10.1136/bmjqs-2017-006813 29146681

[29] Powell C , Tomlinson J , Quinn C , Fylan B . Interventions for self‐management of medicines for community‐dwelling people with dementia and mild cognitive impairment and their family carers: a systematic review. Age Ageing. 2022;51(5):afac089. 10.1093/ageing/afac089 35639800 PMC9154223

[30] Hilmer SN , Gnjidic D . Prescribing for frail older people. Aust Prescr. 2017;40(5):174‐178. 10.18773/austprescr.2017.055.31 29109600 PMC5662436

[31] Tecklenborg S , Byrne C , Cahir C , Brown L , Bennett K . Interventions to reduce adverse drug event‐related outcomes in older adults: a systematic review and meta‐analysis. Drugs Aging. 2020;37(91‐98):91‐98. 10.1007/s40266-019-00738-w 31919801

[32] King VJ , Stevens A , Nussbaumer‐Streit B , Kamel C , Garritty C . Paper 2: performing rapid reviews. Syst Rev. 2022;11(1):151. 10.1186/s13643-022-02011-5 35906677 PMC9338520

[33] Khangura S , Konnyu K , Cushman R , Grimshaw J , Moher D . Evidence summaries: the evolution of a rapid review approach. Syst Rev. 2012;1:10. 10.1186/2046-4053-1-10 22587960 PMC3351736

[34] Garritty C , Gartlehner G , Nussbaumer‐Streit B , et al. Cochrane rapid reviews methods group offers evidence‐informed guidance to conduct rapid reviews. J Clin Epidemiol. 2021;130:13‐22. 10.1016/j.jclinepi.2020.10.007 33068715 PMC7557165

[35] Tomlinson J , Cheong VL , Fylan B , et al. Successful care transitions for older people: a systematic review and meta‐analysis of the effects of interventions that support medication continuity. Age Ageing. 2020;49(4):558‐569. 10.1093/ageing/afaa002 32043116 PMC7331096

[36] Page MJ , McKenzie JE , Bossuyt PM , et al. The PRISMA 2020 statement: an updated guideline for reporting systematic reviews. BMJ (Clinical Research Ed.). 2021;372(71):71. 10.1136/bmj.n71 PMC8005924 33782057

[37] Hollnagel E . RAG—The Resilience Analysis Grid. Ashgate Publishing Limited; 2011.

[38] Moons P , Goossens E , Thompson DR . Rapid reviews: the pros and cons of an accelerated review process. Eur J Cardiovasc Nurs. 2021;20(5):515‐519. 10.1093/eurjcn/zvab041 34007994

[39] Bernsten C , Björkman I , Caramona M , et al. Improving the well‐being of elderly patients via community pharmacy‐based provision of pharmaceutical care: a multicentre study in seven European countries. Drugs Aging. 2001;18(1):63‐77. 10.2165/00002512-200118010-00005 11232739

[40] Sturgess IK , McElnay JC , Hughes CM , Crealey G . Community pharmacy based provision of pharmaceutical care to older patients. Pharm World Sci. 2003;25(5):218‐226. 10.1023/a:1025860402256 14584229

[41] Akers JL , Meer G , Kintner J , Shields A , Dillon‐Sumner L , Bacci JL . Implementing a pharmacist‐led in‐home medication coaching service via community‐based partnerships. J Am Pharm Assoc. 2019;59(2):243‐251. 10.1016/j.japh.2018.11.008 30638730

[42] Benoit M‐L . Medication adherence and safety program for community‐dwelling seniors with chronic conditions. J Doctoral Nurs Pract. 2016;9(2):170‐176. 10.1891/2380-9418.9.2.170 32750985

[43] Bilotta C , Lucini A , Nicolini P , Vergani C . An easy intervention to improve short‐term adherence to medications in community‐dwelling older outpatients. A pilot non‐randomised controlled trial. BMC Health Serv Res. 2011;11:158. 10.1186/1472-6963-11-158 21729274 PMC3146408

[44] Fulmer TT , Feldman PH , Kim TS , et al. An intervention study to enhance medication compliance in community‐dwelling elderly individuals. J Gerontol Nurs. 1999;25(8):6‐9. 10.3928/0098-9134-19990801-04 10711101

[45] Griffiths R , Johnson M , Piper M , Langdon R . A nursing intervention for the quality use of medicines by elderly community clients. Int J Nurs Pract. 2004;10(4):166‐176. 10.1111/j.1440-172X.2004.00476.x 15265227

[46] Hayes TL , Cobbinah K , Dishongh T , et al. A study of medication‐taking and unobtrusive, intelligent reminding. Telemed e‐Health. 2009;15(8):770‐776. 10.1089/tmj.2009.0033 PMC2998278 19780692

[47] Holden RJ , Campbell NL , Abebe E , et al. Usability and feasibility of consumer‐facing technology to reduce unsafe medication use by older adults. Res Soc Admin Pharm. 2020;16(1):54‐61. 10.1016/j.sapharm.2019.02.011 PMC6710164 30853507

[48] Insel KC , Cole L . Individualizing memory strategies to improve medication adherence. Appl Nurs Res. 2005;18(4):199‐204. 10.1016/j.apnr.2004.08.007 16298695

[49] Insel KC , Einstein GO , Morrow DG , Koerner KM , Hepworth JT . Multifaceted prospective memory intervention to improve medication adherence. J Am Geriatr Soc. 2016;64(3):561‐568. 10.1111/jgs.14032 27000329 PMC4806399

[50] Lagerin A , Carlsson AC , Nilsson G , Westman J , Törnkvist L . District nurses’ preventive home visits to 75‐year‐olds: an opportunity to identify factors related to unsafe medication management. Scand J Public Health. 2014;42(8):786‐794. 10.1177/1403494814550680 25260640

[51] LeBlanc RG , Choi J . Optimizing medication safety in the home. Home Healthcare Now. 2015;33(6):313‐319. 10.1097/NHH.0000000000000246 26034822

[52] Lenaghan E , Holland R , Brooks A . Home‐based medication review in a high risk elderly population in primary care ‐ the POLYMED randomised controlled trial. Age Ageing. 2007;36(3):292‐297. 10.1093/ageing/afm036 17387123

[53] Martin D , Kripalani S , DuRapau VJ . Improving medication management among at‐risk older adults. J Gerontol Nurs. 2012;38(6):24‐34. 10.3928/00989134-20120509-01 PMC3785231 22587641

[54] Martin BA , Chewning BA , Margolis AR , Wilson DA , Renken J . Med wise: a theory‐based program to improve older adults’ communication with pharmacists about their medicines. Res Soc Admin Pharm. 2016;12(4):569‐577. 10.1016/j.sapharm.2015.09.010 26508269

[55] Meyer M , Enguidanos S , Zhu Y , Likar D , Batra R . Community Medication Education, Data, & Safety (C‐MEDS): findings from a pilot project. J Am Geriatr Soc. 2021;69(3):813‐821. 10.1111/jgs.16981 33355939

[56] Miller MJ , Abrams MA , Barbara M , et al. Promoting health communication between the community‐dwelling well‐elderly and pharmacists: the Ask Me 3 program. J Am Pharm Assoc. 2008;48(6):784‐792. 10.1331/JAPhA.2008.07073 19019809

[57] Stewart RB , Murray MD , Birt JA , Manatunga AK , Darnell JC . Medication compliance in elderly outpatients using twice‐daily dosing and unit‐of‐use packaging. Ann Pharmacother. 1993;27(5):616‐621. 10.1177/106002809302700517 8347915

[58] Neafsey PJ , Strickler Z , Shellman J , Padula AT . Delivering health information about self‐medication to older adults: use of touchscreen‐equipped notebook computers. J Gerontol Nurs. 2001;27(11):19‐27. 10.3928/0098-9134-20011101-08 11820354

[59] Park E , Kim J . The impact of a nurse‐led home visitation program on hypertension self‐management among older community‐dwelling Koreans. Public Health Nurs. 2016;33(1):42‐52. 10.1111/phn.12220 26250719

[60] Park M . Effects of interactive pictorial education on community dwelling older adult's self efficacy and knowledge for safe medication. J Korean Acad Nurs. 2011;41(6):795‐804. 10.4040/jkan.2011.41.6.795 22310864

[61] Parker R , Frampton C , Blackwood A , Shannon A , Moore G . An electronic medication reminder, supported by a monitoring service, to improve medication compliance for elderly people living independently. J Telemed Telecare. 2012;18(3):156‐158. 10.1258/jtt.2012.SFT108 22362828

[62] Poureslami I , Kwan S , Lam S , Khan N , FitzGerald JM . Assessing the effect of culturally specific audiovisual educational interventions on attaining self‐management skills for chronic obstructive pulmonary disease in Mandarin‐ and cantonese‐speaking patients: a randomized controlled trial. Int J Chronic Obstruct Pulm Dis. 2016;11:1811‐1822. 10.2147/COPD.S105408 PMC4976815 27536093

[63] Schulz RM , Porter C , Lane M , Cornman C , Branham L . Impact of a medication management system on nursing home admission rate in a community‐dwelling nursing home–eligible Medicaid population. Am J Geriatr Pharmacother. 2011;9(1):69‐79. 10.1016/j.amjopharm.2011.02.008 21459310

[64] Sidel VW , Beizer JL , Lisi‐Fazio D , et al. Controlled study of the impact of educational home visits by pharmacists to high‐risk older patients. J Community Health. 1990;15(3):163‐174. 10.1007/BF01350254 2195066

[65] Suzuki R , Hasegawa T . Evaluation of a one‐dose package medication support system for community‐based elderly patients and eldercare facilities. Am J Health Syst Pharm. 2018;75(9):e202‐e212. 10.2146/ajhp170176 29691263

[66] Wang CJ , Fetzer SJ , Yang YC , Wang JJ . The impacts of using community health volunteers to coach medication safety behaviors among rural elders with chronic illnesses. Geriatr Nurs. 2013;34(2):138‐145. 10.1016/j.gerinurse.2012.12.013 23414637

[67] Whittaker CF , Tom SE , Bivens A , Klein‐Schwartz W . Evaluation of an educational intervention on knowledge and awareness of medication safety in older adults with low health literacy. Am J Health Educ. 2017;48(2):100‐107. 10.1080/19325037.2016.1271754

[68] Wong AKC , Wong FKY , Chang K . Effectiveness of a community‐based self‐care promoting program for community‐dwelling older adults: a randomized controlled trial. Age Ageing. 2019;48(6):852‐858. 10.1093/ageing/afz095 31437272

[69] Poonprapai P , Lerkiatbundit S , Saengcharoen W . Family support‐based intervention using a mobile application provided by pharmacists for older adults with diabetes to improve glycaemic control: a randomised controlled trial. Int J Clin Pharm. 2022;44(3):680‐688. 10.1007/s11096-022-01389-5 35247147

[70] Zhang S , Zhu D , Qi Z , et al. Effects of home medication review on drug‐related problems and health‐related quality of life among community‐dwelling older adults in China. J Am Pharm Assoc. 2022;62(2):481‐486. 10.1016/j.japh.2021.10.023 34776338

[71] Falamić S , Lucijanić M , Ortner‐Hadžiabdić M , Marušić S , Bačić‐Vrca V . Pharmacists’ interventions improve health‐related quality of life of rural older person on warfarin: a randomized controlled trial. Sci Rep. 2021;11(1):21897. 10.1038/s41598-021-01394-0 34754004 PMC8578616

[72] Lawton R , O'Hara JK , Sheard L , et al. Can patient involvement improve patient safety? A cluster randomised control trial of the Patient Reporting and Action for a Safe Environment (PRASE) intervention. BMJ Qual Saf. 2017;26(8):622‐631. 10.1136/bmjqs-2016-005570 PMC5537521 28159854

[73] Murray J , Hardicre N , Birks Y , O′Hara J , Lawton R . How older people enact care involvement during transition from hospital to home: a systematic review and model. Health Expect. 2019;22(5):883‐893. 10.1111/hex.12930 31301114 PMC6803411

[74] Hibbard JH , Stockard J , Mahoney ER , Tusler M . Development of the Patient Activation Measure (PAM): conceptualizing and measuring activation in patients and consumers: development of the Patient Activation Measure (PAM). Health Serv Res. 2004;39(4p1):1005‐1026. 10.1111/j.1475-6773.2004.00269.x 15230939 PMC1361049

[75] O′Hara JK , Baxter R , Hardicre N . ‘Handing over to the patient’: a FRAM analysis of transitional care combining multiple stakeholder perspectives. Appl Hergon. 2020;85:103060. 10.1016/j.apergo.2020.103060 32174348

[76] Wilson E , Hm Chen A , Grumbach K , Wang F , Fernandez A . Effects of limited English proficiency and physician language on health care comprehension. J Gen Intern Med. 2005;20:800‐806. 10.1111/j.1525-1497.2005.0174.x 16117746 PMC1490205

[77] Wade C , Malhotra AM , McGuire P , Vincent C , Fowler A . Action on patient safety can reduce health inequalities. BMJ (Clinical Research Ed.). 2022;376:e067090. 10.1136/bmj-2021-067090 PMC9731338 35351684

[78] O′Hara JK , Aase K , Waring J . Scaffolding our systems? Patients and families ‘reaching in’ as a source of healthcare resilience. BMJ Qual Saf. 2019;28(1):3‐6. 10.1136/bmjqs-2018-008216 29764929

[79] Silcock J , Marques I , Olaniyan J , et al. Co‐designing an intervention to improve the process of deprescribing for older people living with frailty in the United Kingdom. Health Expect. 2023;26(1):399‐408. 10.1111/hex.13669 36420768 PMC9854320

[80] Alharbi K , van Marwijk H , Reeves D , Blakeman T . Identification and management of frailty in English primary care: a qualitative study of national policy. BJGP Open. 2020;4(1):bjgpopen20X101019. 10.3399/bjgpopen20X101019 PMC7330193 32184213

[81] Swinglehurst D , Fudge N . Organising polypharmacy: unpacking medicines, unpacking meanings‐an ethnographic study. BMJ Open. 2021;11(8):e049218. 10.1136/bmjopen-2021-049218 PMC8395269 34446490

[82] Eton D , Ramalho de Oliveira D , Egginton J , et al. Building a measurement framework of burden of treatment in complex patients with chronic conditions: a qualitative study. Patient Relat Outcome Meas. 2012;3:39‐49. 10.2147/PROM.S34681 23185121 PMC3506008

[83] Hounkpatin HO , Roderick P , Morris JE , et al. Change in treatment burden among people with multimorbidity: protocol of a follow up survey and development of efficient measurement tools for primary care. PLoS One. 2021;16(11):e0260228. 10.1371/journal.pone.0260228 34843541 PMC8629211

[84] Nahum‐Shani I , Smith SN , Spring BJ , et al. Just‐in‐time adaptive interventions (JITAIs) in mobile health: key components and design principles for ongoing health behavior support. Ann Behav Med. 2018;52(6):446‐462. 10.1007/s12160-016-9830-8 27663578 PMC5364076

[85] Ofcom . Digital exclusion. A review of Ofcom's research on digital exclusion among adults in the UK. 2022. Accessed February 2, 2023. https://www.ofcom.org.uk/__data/assets/pdf_file/0022/234364/digital-exclusion-review-2022.pdf

[86] Slattery P , Saeri AK , Bragge P . Research co‐design in health: a rapid overview of reviews. Health Res Policy Syst. 2020;18(1):17. 10.1186/s12961-020-0528-9 32046728 PMC7014755

[87] May CR , Eton DT , Boehmer K , et al. Rethinking the patient: using Burden of Treatment Theory to understand the changing dynamics of illness. BMC Health Serv Res. 2014;14:281. 10.1186/1472-6963-14-281 24969758 PMC4080515

[88] May C , Montori VM , Mair FS . We need minimally disruptive medicine. BMJ. 2009;339:b2803. 10.1136/bmj.b2803 19671932

[89] Cross AJ , Elliott RA , Petrie K , Kuruvilla L , George J . Interventions for improving medication‐taking ability and adherence in older adults prescribed multiple medications. Cochrane Database Syst Rev. 2020;5(5):CD012429. 10.1002/14651858.CD012419.pub2 PMC7207012 32383493

