# Supplementary material for: A rapid review of interventions to improve medicine self‐management for older people living at home
Source: Health Expect. 2023 Mar 14;26(3):945–88. doi: 10.1111/hex.13729 (PMC10154809; doi:10.1111/hex.13729)
Supplement: Supplementary file 1 — Supporting information. [file HEX-26--s001.docx]

# Appendix 1: MeSH terms and Key words

| **Pubmed search strings, up to April 2022** | |
| --- | --- |
| **Concept** | **String** |
| Frail older people | "Frailty"[MeSH Terms] OR aged[mh] OR "Aging"[MeSH Terms] OR "Frail Elderly"[MeSH Terms] OR "Multiple Chronic Conditions"[MeSH Terms] OR "Multimorbidity"[MeSH Terms] OR ("medic*"[All Fields] AND "packag*"[All Fields]) OR ("tablet*"[All Fields] AND "split*"[All Fields]) OR ("tablet*"[All Fields] AND "break*"[All Fields]) OR ("syring*"[All Fields] OR "self inject*"[All Fields]) OR ("difficult*"[All Fields] AND "swallow*"[All Fields] AND "medic*"[All Fields]) |
| Self-managing | (((((("empowerment"[All Fields]) OR ("participatory"[All Fields])) OR ("manage disease"[All Fields])) OR ("medication management"[All Fields])) OR ("morbidity management"[All Fields])) OR ("disease management"[All Fields])) OR ("Self-Management"[MeSH Terms] OR "Self Care"[MeSH Terms] OR "Patient Education as Topic"[MeSH Terms] OR "Self Efficacy"[MeSH Terms] OR "Disease Management"[MeSH Terms] OR "medication therapy management/education"[MeSH Terms] OR "medication therapy management/organization and administration"[MeSH Terms] OR "Independent Living"[MeSH Terms]) |
| Medications | ((((((("Polypharmacy" [Mesh] OR "Medication Adherence" [Mesh] OR "Medication Errors" [Mesh] OR "Drug Interactions" [Mesh] OR "Drug-Related Side Effects and Adverse Reactions" [Mesh] OR "Health Communication" [Mesh] OR "Patient Medication Knowledge"[Mesh]) OR ("Drug Administration Routes"[Mesh])) OR ((((((((((((((("complex medication regimens"[All Fields]) OR ("complex medication regimen"[All Fields])) OR ("medication safety"[All Fields])) OR ("medicine safety"[All Fields]) ) OR ("medication adherence"[All Fields])) OR ("treatment adherence"[All Fields])) ) OR ("nonadherence"[All Fields]))) OR ("medication compliance"[All Fields])) OR ("medication error"[All Fields])) OR ("medication problem"[All Fields])) OR ("medication problems"[All Fields])) OR ("medicine errors"[All Fields])) OR ("medicine problems"[All Fields]))) OR ((medic* AND ((error manag*) OR (error resol*))))) OR (("medication supply"[All Fields]) OR (medication* supply patient*)OR (communicat* patient* medicat*) OR (medicat* support*))) OR (("medication discrepancy"[All Fields]) OR (discordan* medic*))) OR (((("Nurses"[Mesh] OR "Medical Staff"[Mesh] OR "General Practitioners"[Mesh] OR "Pharmacists"[Mesh]) AND (patient* medic*)) OR (("Nurses"[Mesh] OR "Medical Staff"[Mesh] OR "General Practitioners"[Mesh] OR "Pharmacists"[Mesh]) AND (medic* support*))) OR (("Nurses"[Mesh] OR "Medical Staff"[Mesh] OR "General Practitioners"[Mesh] OR "Pharmacists"[Mesh]) AND (communicat* medic*))) |
| At home with/without family carer | ((("Caregivers"[MeSH Terms]) OR ((((((("relative"[All Fields]) ) ) OR ("partner"[All Fields])) OR ("spouse"[All Fields])) OR ("sibling"[All Fields])) OR ("couple"[All Fields]))) OR ((("living at home"[All Fields]) OR ("living in the community"[All Fields])) OR ("community dwelling"[All Fields]))) OR ("child"[All Fields]) |

| **EMBASE search strings, up to April 2022** | |
| --- | --- |
| **Concept** | **String** |
| Frail older people | 'frailty'/exp OR 'aged'/exp OR 'aging'/exp OR 'frail elderly'/exp OR 'multiple chronic conditions'/exp OR 'drug packaging'/exp OR (tablet* NEAR/3 split*) OR (tablet* NEAR/3 break*) OR (tablet* NEAR/3 cut*) OR ((difficult* NEAR/4 swallow*) AND medic*) OR 'syringe'/exp OR 'self injection'/exp |
| Self-managing | ('self care'/exp OR ('patient eduction' AND 'medication'/exp) OR 'medication therapy management'/exp OR 'independent living'/exp OR 'empowerment'/exp OR 'disease management'/exp OR participatory) |
| Medications | 'polypharmacy'/exp OR 'medication compliance'/exp OR 'medication error'/exp OR 'drug interaction'/exp OR 'adverse drug reaction'/exp OR 'drug administration route'/exp OR 'patient education'/exp OR (('medication'/exp OR medication) AND regimen AND ('complexity'/exp OR complexity)) OR (('medication'/exp OR medication) AND ('safety'/exp OR safety)) OR 'medication discrepancy'/exp OR 'medication discrepancy' OR (('medication'/exp OR medication) AND ('discordance'/exp OR discordance)) OR (manag* AND ('error'/mj/exp OR 'error'/exp OR 'error') AND ('medication'/mj/exp OR 'medication'/exp OR 'medication')) OR (('medication'/exp OR medication) AND ('error'/exp OR error) AND ('resolution'/exp OR resol*)) OR (('medication'/exp OR medication) AND ('error'/exp OR error)) OR (('medication'/exp OR medication) AND problem*) OR (('patient'/exp OR 'patient') AND ('medication'/exp OR 'medication') AND supply) OR (('nurse'/exp OR 'health care personnel'/exp OR 'physician'/exp OR 'general practitioner'/exp OR 'pharmacist'/exp) AND ('medication'/exp OR medication) AND ('support'/exp OR 'support') AND ('medication'/exp OR 'medication')) OR (('nurse'/exp OR 'health care personnel'/exp OR 'physician'/exp OR 'general practitioner'/exp OR 'pharmacist'/exp) AND ('medication'/exp OR medication) AND ('communication'/exp OR communication)) OR (('nurse'/exp OR 'health care personnel'/exp OR 'physician'/exp OR 'general practitioner'/exp OR 'pharmacist'/exp) AND ('patient'/exp OR 'patient') AND ('medication'/exp OR 'medication')) |
| At home with/without family carer | 'relative'/exp OR 'spouse'/exp OR 'child'/exp OR 'partner'/exp OR 'sibling'/exp OR 'couple'/exp OR (('family'/exp OR family) AND ('carer'/exp OR care*)) OR (informal AND ('care'/exp OR care*)) OR (('community'/exp OR community) AND dwelling) OR 'community dwelling elderly' OR 'caregiver'/exp OR ((home OR communit*) NEAR/1 (dwell* OR liv*)) |
