# Supplementary material for: A rapid review of interventions to improve medicine self‐management for older people living at home
Source: Health Expect. 2023 Mar 14;26(3):945–88. doi: 10.1111/hex.13729 (PMC10154809; doi:10.1111/hex.13729)
Supplement: Supplementary file 2 — Supporting information. [file HEX-26--s002.docx]

# Appendix 2: Full text screening checklist

| **PICO** | **Inclusion Criteria** | **Exclusion Criteria** |
| --- | --- | --- |
| Population | - Aged 65 and over living at home - Manage medications independently - Manage medications supported by informal carers | - Sample does not include people 65 and above - People in sample live in care facilities - People in sample are in hospital at the time of the study - People live at home but medications are managed by a paid carer or other professional |
| Intervention’s aim | - To enhance self-management of health condition (e.g. monitoring symptoms and respond to changes) in patient and/or support network including adherence, error management, and supply management - To  improve communication between patient (and/or support network) and healthcare providers about medicines | - Aimed to de-prescribe medicines only |
| Intervention timeline | - Has already been delivered - Feasibility study | - Protocol intervention study |
| Target of intervention | - Aimed at patients and or support network | - Aimed at healthcare professionals |
| Intervention type and specifications | - Educational - Coaching - Training - Empowerment - Medication review with a patient facing component - Medicines optimisation programme with a patient-facing component - Self-help activities run by patients - Based on the use of Information Communication Technology  (apps, internet portals, customized texts, follow up phone calls) | - Focused just on the management of a condition |
| Outcomes | - Improved patient safety - Decreased hospitalization - Increased self-management skills in patients and/or support network - Increased self-confidence in patients and/or support network - Increased involvement of patients around safety in primary care - Improvements in the management of medication systems, including at transitions |  |
